# High turnover in electro-oxidation of alcohols and ethers with a glassy carbon-supported phenanthroimidazole mediator†
†Electronic supplementary information (ESI) available. See DOI: 10.1039/c7sc02482g



**DOI:** 10.1039/c7sc02482g

**Published:** 2017-07-17

**Authors:** Bruce M. Johnson, Robert Francke, R. Daniel Little, Louise A. Berben

**Affiliations:** a Department of Chemistry , University of California , Davis , CA 95616 , USA . Email: laberben@ucdavis.edu; b Institut für Chemie , Abteilung Technische Chemie , Universität Rostock , Germany . Email: robert.francke@uni-rostock.de; c Department of Chemistry and Biochemistry , University of California , Santa Barbara , CA 93106 , USA . Email: little@chem.ucsb.edu

## Abstract

Glassy carbon electrodes covalently modified with a phenanthroimidazole mediator promote electrochemical alcohol and ether oxidation: three orders of magnitude increase in TON, to ∼15 000 in each case, was observed compared with homogeneous mediated reactions. We propose the deactivation pathways in homogeneous solution are prevented by the immobilization: modified electrode reversibility is increased for a one-electron oxidation reaction. The modified electrodes were used to catalytically oxidize *p*-anisyl alcohol and 1-((benzyloxy)methyl)-4-methoxybenzene, selectively, to the corresponding benzaldehyde and benzyl ester, respectively.

## Abstract 2

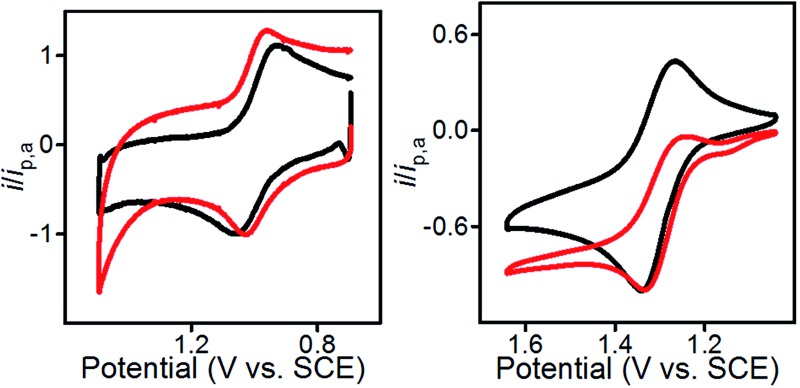
Glassy carbon electrodes covalently modified with a phenanthroimidazole mediator promote electrochemical alcohol and ether oxidation: three orders of magnitude increase in TON, to ∼15 000 in each case, was observed compared with homogeneous mediated reactions.

## Introduction

Chemically modified electrodes are potential candidates for the oxidation and reduction reactions associated with fuel cells. In particular, methods for the oxidation of biologically-derived alternative fuels would enable their use in renewable energy applications: these substrates include electron rich organic alcohols and ethers which are derived from the degradation of lignin. Concurrent with a need for fuel cell chemistry, the value of organic redox mediators has become more apparent as the scientific community works towards establishing greener practices in synthetic organic chemistry. When electric current is used as a terminal oxidant for synthetic oxidation reactions, toxic and dangerous sacrificial (stoichiometric) chemical oxidants and copious waste products can be eliminated.^1,2^ In this work we employ the recent advances in organic mediator chemistry in the development of a robust chemically modified electrode system. We demonstrate advances in performance in the field of mediated organic electro-oxidation, and we do this using carbon-based electrodes suitable for application in fuel cells based on biologically-derived alcohols and ethers.

**Scheme 1:**
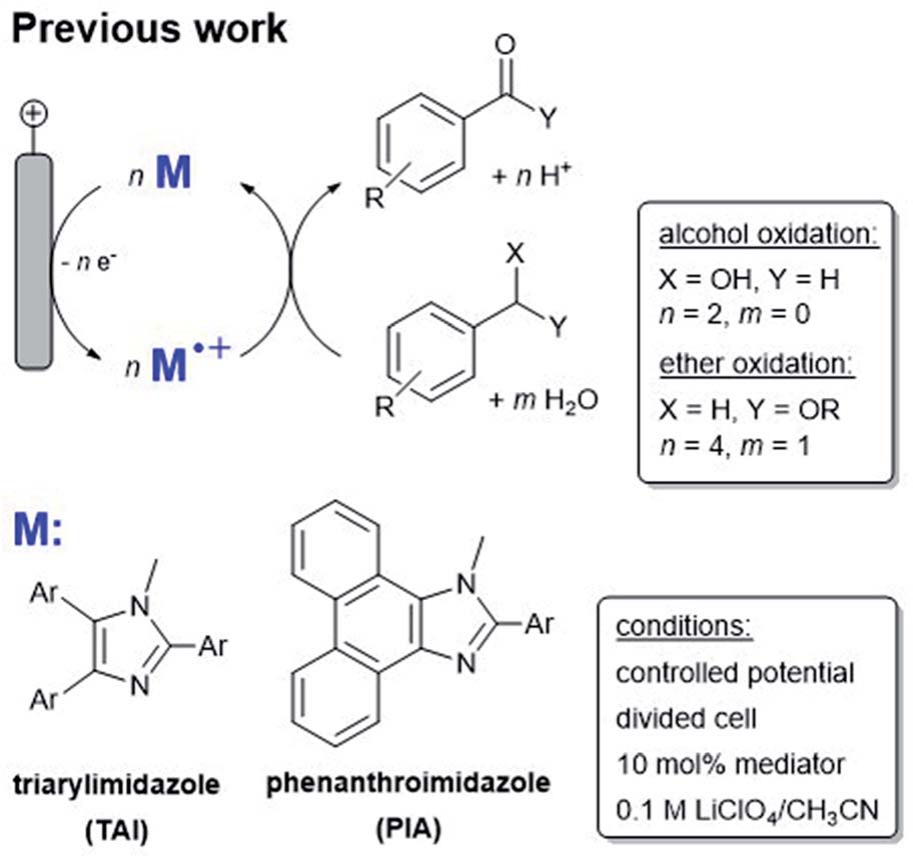
(Top) Oxidation of alcohols by arylimidazole mediators (M) in homogeneous solution. (Bottom) Examples of arylimidazole mediator classes TAI and PIA.

Mediated electrochemical reactions employ a redox active shuttle that enables reduced electrode poisoning, improved electron transfer kinetics and reaction selectivity compared with the direct electrochemical oxidation or reduction of organic substrates. Mediated electrochemical reactions also offer the ability to dial in a chosen redox potential for a desired reaction by substituting electron donating or withdrawing groups on the substrate.^3^ Recently, a new class of metal-free, easy to synthesize redox mediators based on the arylimidazole framework was developed (Scheme 1).

To date, these organic mediators have been useful tools in processes including C–H bond activation, alcohol oxidation and epoxide ring opening reactions,^4a–d^ but a key weakness of arylimidazole mediators is the relatively poor stability of the activated (oxidized) form and consequently, the necessity for employing large quantities (typically at least 10 mol%). Homogeneous mediated reactions also require a divided electrolysis cell, a configuration that is not ideal for scale-up. Current understanding surrounding mediator instability is that intermediate oxidation states on the arylimidazole have radical ionic character, necessary to promote one electron chemistry,^5^ and this enables decomposition pathways that likely involve radical cation dimer formation.^6–8^ We reasoned that immobilization of a mediator on an electrode surface could prevent dimer formation and enhance stability; immobilization of mediators could also provide a route for their incorporation into fuel cells. In contrast to the rich electrode modification chemistry explored with organometallic electrocatalysts,^9a–h^ the study of immobilized organic mediators and their application in organic synthesis is limited.^4a,10a–c^

To explore the possibility for improving the properties of organic mediators by surface attachment, we studied the phenanthroimidazole mediator (PIA, see Scheme 1), which is a congener of the arylimidazole family, previously reported by Little and co-workers in homogeneous solution (Scheme 1, bottom).^4*c*^ In those reports a variety of benzyl alcohol and ether substrates were oxidized by a series of PIA derivatives. Substrates of this nature are of particular interest as lignin models and functional subunits.^11a–c^ Lignin is a large component of cell walls in plants and a target carbon feedstock if it can be harnessed efficiently. Mediators capable of oxidizing electron donor-functionalized benzyl alcohols and ethers are candidates for lignin oxidative disassembly.

## Results and discussion

**Scheme 2:**
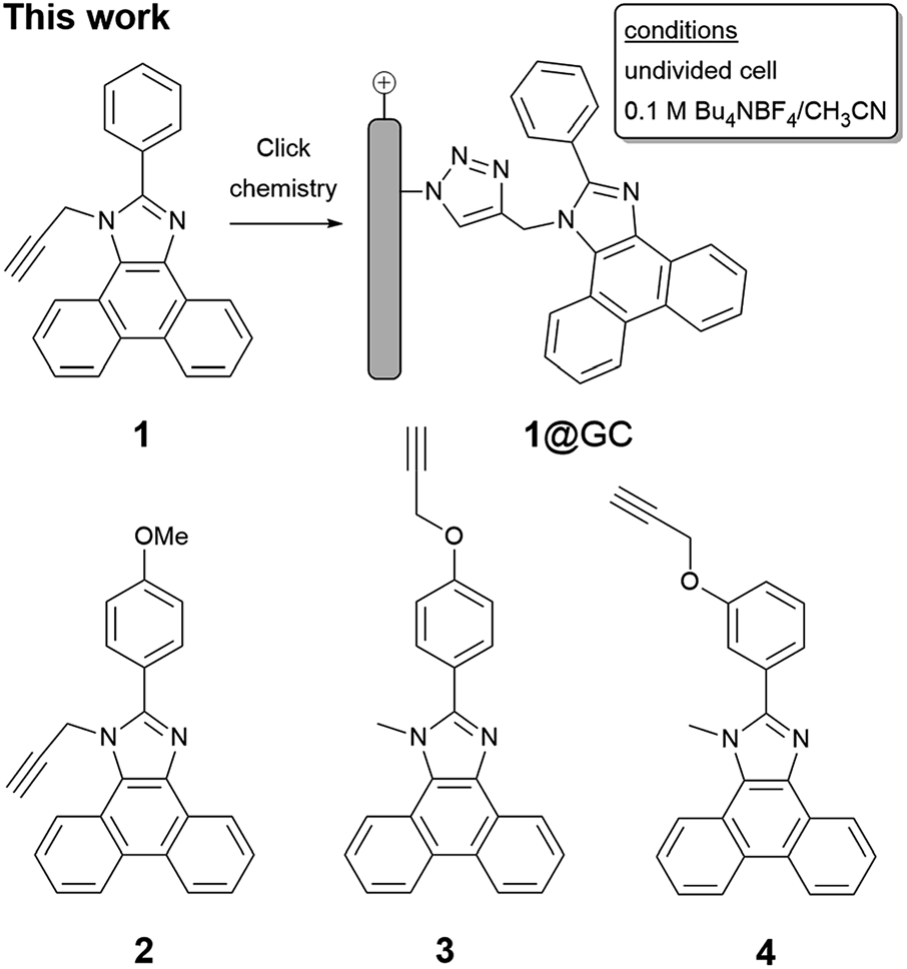
(Top) Covalent modification of GC with **1**. (Bottom) Line drawings of **2–4**.

To enable immobilization of a PIA mediator, we prepared mediators where either the *N*-methyl group was replaced with propargyl, to afford **1** and **2**, or the phenyl ring was functionalized with a propargyloxy group, as in **3** and **4** (Scheme 2 and Fig. S1–S8†). A copper catalyzed cycloaddition reaction of **1–4** with azide-modified glassy carbon (GC) produced a covalently decorated electrode that was used for further study.^9a,12^ The modified electrodes are labelled as **1**@GC – **4**@GC throughout and complete experimental details describing their preparation are given in the ESI.†

**Fig. 1:**
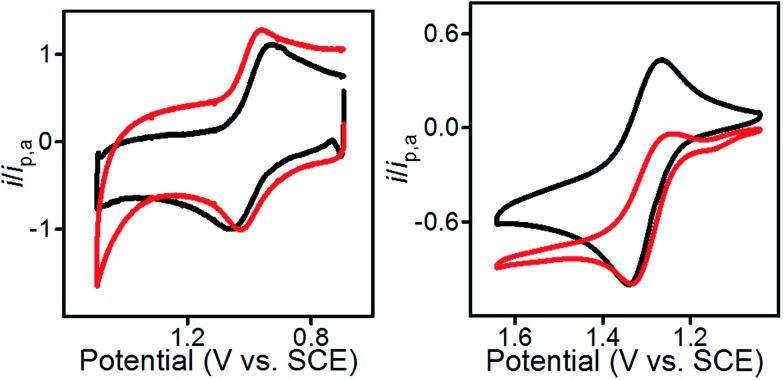
(Left) CVs of **1**@GC; (Right) CVs of homogeneous solution of **1**. All CVs recorded in 0.1 M Bu_4_NBF_4_ MeCN solution at 10 (red) and 100 (black) mV s^–1^.

Characterization of the modified electrodes immersed in a 0.1 M Bu_4_NBF_4_–MeCN solution, using cyclic voltammetry (CV), showed reversible oxidation events that occur between *E*_1/2_ = +1.00 V and +1.05 *vs.* SCE for all four materials, **1**@GC – **4**@GC (Fig. 1, S9† and Table 1). The reversibility of the one-electron oxidation processes is underscored by the ratio of the oxidative peak current (*i*_pa_) to reductive peak current (*i*_pc_), given by *i*_pa_/*i*_pc_. For **1**@GC through **4**@GC, *i*_pa_/*i*_pc_ ratios are all very close to 1.0, and fall between 0.93 and 1.1 for scans collected at 100 mV s^–1^ (Table 1). The reversibility of the one-electron oxidation event suggests that there is limited decomposition of the mediator upon oxidation.

**Table 1:** Electrochemical parameters of **1–4** in homogeneous solution and on GC

	*E*_1/2_@GC (V *vs.* SCE)	*E*_1/2_ solution (V *vs.* SCE)	*i*_pa_/*i*_pc_ ^*a*^@GC	*i*_pa_/*i*_pc_ ^*a*^ solution
**1**	1.00	1.31	0.93, 1.05	1.27, 1.23
**2**	1.04	1.20	0.95, 1.08	1.62, 1.15
**3**	1.05	1.24	1.12, 1.04	1.40, 1.17
**4**	1.04	1.16	1.23, 0.97	1.36, 1.30

^*a*^For data collected at 100 and 10 mV s^–1^, respectively.

A comparison of **1**@GC – **4**@GC with CVs for **1–4**, collected as homogeneous 1.0 mM solutions in 0.1 M Bu_4_NBF_4_ MeCN, further illustrates the robustness of the modified electrode assemblies and the enhanced stability of the one-electron oxidized species, such as [**1**@GC]^+^˙, over homogeneous species such as **1** ^+^˙ (Fig. 1 and S10†). At 100 mV s^–1^ values of *i*_pa_/*i*_pc_ for the homogeneous mediators range from 1.27–1.62 (Table 1). In all cases, the value of *i*_pa_/*i*_pc_ is between 0.1 and 0.6 units higher than the corresponding value for mediator immobilized on GC. The lack of complete reversibility for the homogeneous mediators is further emphasized using a comparison of CVs collected at 10 and 100 mV s^–1^ which we plotted with the anodic current response normalized to *i*_pa_ for 100 mV s^–1^ (Fig. 1 and S10†). CVs for homogeneous **1** collected at 10 mV s^–1^ show a greater ratio for *i*_pa_/*i*_pc_ consistent with further loss of reversibility at low scan rates (Fig. 1, right). CVs for **1**@GC show little variation in the *i*_pa_/*i*_pc_ between 100 and 10 mV s^–1^ (Fig. 1, left). Previously reported PIA mediators studied in homogeneous solution show a similar decrease in reversibility at 10 mV s^–1^ compared with 100 mV s^–1^.^4*c*^

The peak potential of an irreversible (or not completely reversible) redox reaction is highly dependent on the experimental conditions employed for the CV measurement. This phenomenon also leads to *E*_1/2_ measurements that are not representative of the true thermodynamic *E*_1/2_ value in quasi-reversible and irreversible systems. In the present case, the enhanced reversibility of the surface-confined mediators is manifested in the observed *E*_1/2_ values (Table 1). Taking **1**@GC as an example, *E*_1/2_ is +1.0 V whereas the apparent *E*_1/2_ for homogeneous **1** is anodically shifted by 310 mV to +1.31 V: *i.e.* as the one electron couple becomes more reversible, the oxidation potential shifts cathodically toward the thermodynamic *E*_1/2_ value. Smaller, but noticeable, cathodic shifts are observed for **2–4** (Table 1).^13^ This cathodic shift upon immobilization offers a further advantage over homogeneous systems by providing access to lower energy pathways for oxidation chemistry.

Variable scan rate CV measurements were used, with eqn (1), to determine the surface concentration of mediators on GC:^14a,b^
1
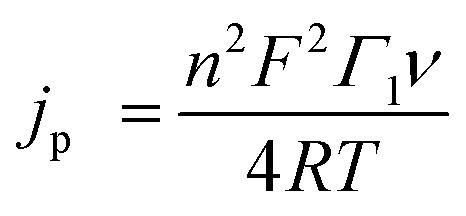



For eqn (1), *j*_p_ is the anodic peak current density in the absence of substrate (A cm^–2^), *n* is the number of electrons involved in the process (*n* = 1), *F* is Faraday's constant (C mol^–1^), *Γ*_1_ is the surface coverage of **1** (mol cm^–1^), and *ν* is the scan rate (V s^–1^). This analysis showed that surface coverage of **1** is 5.5 × 10^–10^ mol cm^–2^ (Table S1 and Fig. S2,† top left), which is comparable to the coverage we observed in control experiments where ferrocene (Fc) was attached to GC (Table S1, Calculation S1 and Fig. S11†), and similar to reports by others concerning the attachment of Fc to GC.^12,15a,b^ Based on the surface concentration of **1**@GC and the dimensions of **1** calculated from typical bond lengths and atomic radii, we estimate that the molecules of **1** have an average spacing of about 2.50 Å between them (Calculation S2†).

**Scheme 3:**
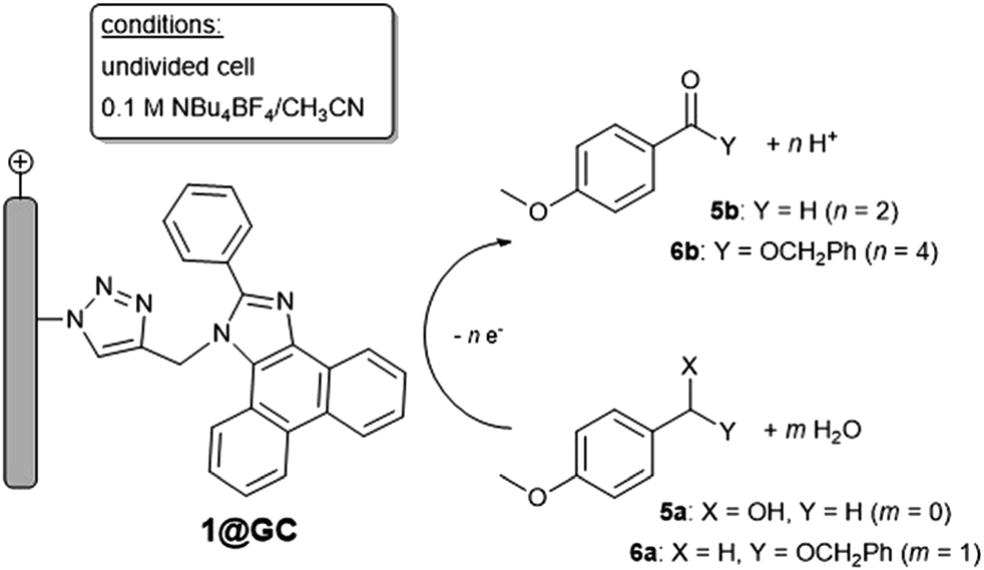
Oxidation of **5a** and **6a** by **1**@GC.

**Fig. 2:**
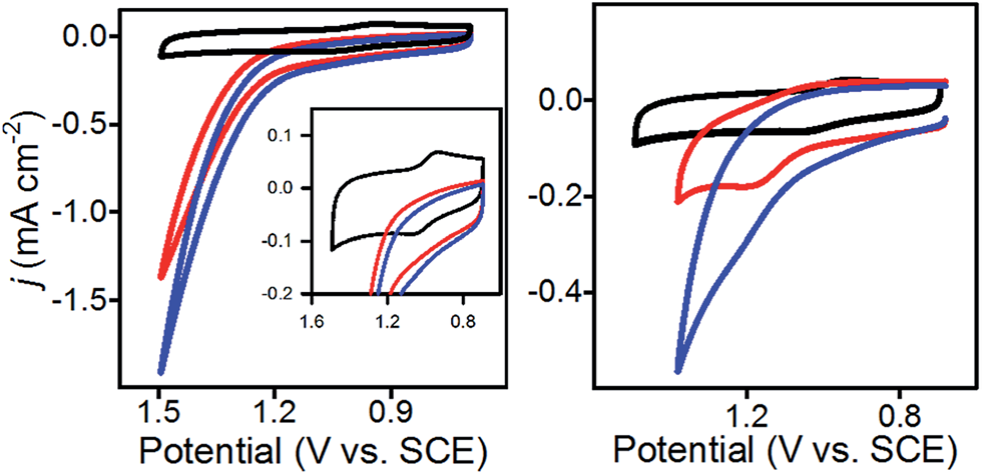
(Left) CVs of (black) **1**@GC, (red) with 20 mM **5a**, 5 mM 2,6-lutidine, and (blue) 20 mM **5a**, 50 mM 2,6-lutidine. Inset: expanded scale. (Right) CVs of (black) **1**@GC (red), **1**@GC with 20 mM **6a**, and (blue) **1**@GC with 20 mM **6a**, 50 mM 2,6-lutidine and 0.1 mL H_2_O. CVs recorded at 100 mV s^–1^ in 0.1 M Bu_4_NBF_4_ MeCN solution.

Mediated catalytic oxidation reactions were initially explored with *p*-anisyl alcohol (*p*-AnOH, **5a**) as substrate using **1**@GC as mediator (Scheme 3). We have chosen 1@GC for in-depth studies since it has the lowest *E*_1/2_: preliminary screens indicate that **1–4** all possess similar reactivity toward substrates. When solutions of **5a** in 0.1 M Bu_4_NBF_4_ MeCN, with added 2,6-lutidine as a base, were scanned using CV in the anodic direction the redox couple associated with **1**@GC was no longer reversible and the current increased 20 fold at 1.37 V *vs.* SCE (Fig. 2, left), an observation that is consistent with observations on homogeneous PIA.^4*c*^

Controlled potential electrolysis (CPE) studies with **5a** and 2,6-lutidine performed over 5 h at 1.37 V *vs.* SCE, revealed 15 000 turnovers to *p*-anisyl aldehyde (**5b**) which was quantified by proton NMR spectroscopy (Fig. S12†). This turnover number (TON) is based on the surface coverage measurement and the amount of product detected after electrolysis (see ESI for details†). During CPE measurements a steady output of charge was observed and the faradaic efficiency (FE) was 78% with regard to generation of **5b**. No products of over-oxidation, such as the carboxylic acid, were observed. We believe that the additional current must go toward electrolyte or solvent degradation. GC-TCD analysis of the headspace did not detect any O_2_ which could potentially form from water. Control CPE experiments in the absence of **1**@GC at the same electrode potential produced no aldehyde. These results demonstrate that the surface-attached mediator affords a 900- or 2300-fold increase in TON compared with earlier work on homogeneous mediated oxidations using PIA or TAI, respectively (Calculation S3†).^4b,c^

We explored the literature and compared **1**@GC to other chemically modified electrodes for electrocatalytic oxidation of alcohols. In 2012 Meyer and co-workers examined the oxidation of benzyl alcohol with a Ru catalyst tethered to TiO_2_. This modified electrode operated at a lower potential (as low as 0.81 V *vs.* SCE) and the highest TON and TOF reported were 2440 and 0.56 s^–1^, respectively.^9*g*^ In 2015, Waymouth and co-workers studied another Ru catalyst tethered at TiO_2_ and its ability to oxidize 2-propanol to acetone. At 0.85 V *vs.* SCE, this performed 14.4 turnovers in 24 hours.^9*f*^ These comparisons further highlight the potential utility of an approach where organic mediators are immobilized: the primary decomposition pathways for organic mediators are intermolecular, and so they can be readily shut down; decomposition pathways for homogeneous inorganic mediators are more varied in their mechanistic details.

The oxidative capabilities of **1**@GC, were also studied with a benzyl ether, 1-((benzyloxy)methyl)-4-methoxybenzene (BMMB, **6a**, Scheme 3). CVs recorded with **1**@GC in the presence of **6a**, 2,6-lutidine and H_2_O produced a 5-fold increase in anodic current at 1.37 V *vs.* SCE and loss of reversibility associated with the one electron oxidation of **1**@GC (Fig. 2, right). After 5 h of electrolysis at +1.37 V *vs.* SCE, the benzyl ester product, **6b** was produced with 70% faradaic efficiency, and TON of 14 000. The ester product was quantified *via* proton NMR (Fig. S13†), and no products of over-oxidation were observed. No ester product was detected for control CPE experiments conducted in the absence of **1**@GC. Comparison to homogeneous mediated reactions previously reported with TAI, show a 1500-fold increase.^4*b*^ Data is not available for the homogeneous PIA system.

To further probe the stability of **1**@GC during oxidation reactions, CVs were recorded before and after electrolysis experiments and these indicated that very little decomposition had occurred (Fig. S14†). Additionally, CPE experiments with **5a** were performed in an undivided cell. After 5 hours of electrolysis the substrate solution was removed and the flask was rinsed, then a second CPE experiment was conducted for 5 hours using the same modified electrode. No significant drop in current density, % conversion to **5b** or TON was observed, further supporting the enhanced stability of the immobilized mediator. The stability of **1**@GC in an undivided cell is a major technological advantage for large scale electrosynthesis: homogeneous systems have previously required divided cell arrangements since the oxidized mediator can diffuse toward the counter electrode where it is deactivated.^4*c*^

Taken together, the results of these experiments, using both CV and CPE measurements, indicate that **1**@GC is longer lived than homogeneous PIA which required rigorous exclusion of light and O_2_ as well as strict regulation of base during electrolysis, or addition of mediator throughout experiments.^4*c*^ At the time of its publication, PIA represented a significant advance in stability compared to the previously developed TAI mediators. Two possible deactivation pathways have been postulated for PIA in homogeneous mediated oxidation reactions. The most likely of these is aggregation: the intermediate PIA˙^+^ molecules can align into a radical “sandwich”, driven by spin pairing.^6,7^ Another possible pairing mechanism is between PIA˙^+^ and PIA, driven by electrostatic interactions.^6,7^ Dimers and oligomers of PIA˙^+^ are less active as oxidants, and immobilization spatially confines mediator molecules to prevent association.

A second possible pathway for deactivation of PIA in homogeneous solution would also be prevented by immobilization: it is possible that PIA˙^+^ is deprotonated in the basic environment provided by excess 2,6-lutidine, leading to the decomposition of the radical. When **1**@GC is employed, the compound is protected within a pH gradient generated by oxidation of the substrate at the anode. The working electrode is held at positive potential, and the local solution is acidified which protects **1**@GC from deprotonation.

**Fig. 3:**
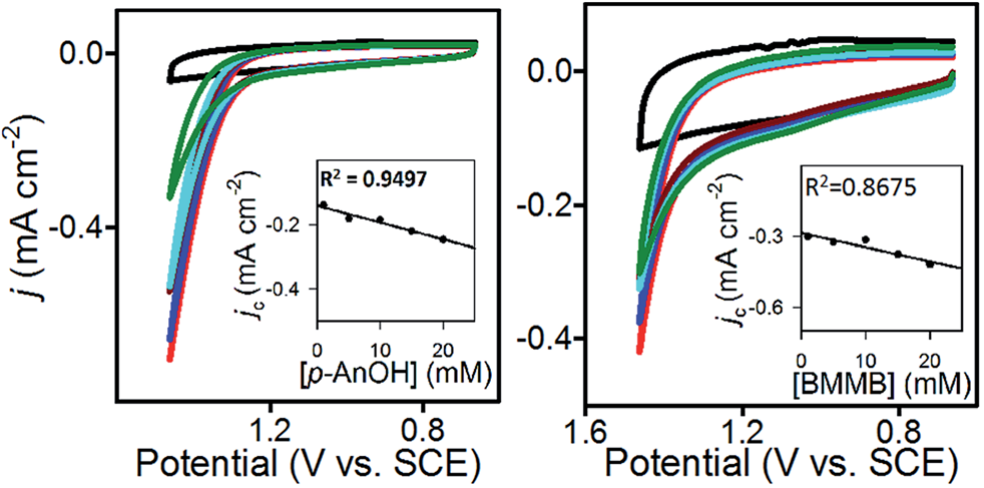
CVs of (black) **1**@GC with (colors) 20 mM, 15 mM, 10 mM, 5 mM and 1 mM substrate (left, **5a**; right, **6a**) and 5 mM 2,6-lutidine. Inset: *j*_c_ *vs.* [substrate] plots. Recorded at 0.05 V s^–1^ in 0.1 M Bu_4_NBF_4_ MeCN. These plots demonstrate that each of the oxidation reactions are first order with respect to substrate.

To further characterize **1**@GC, we determined the rate constants (*k*_cat_) associated with the oxidation of **5a** and **6a** by **1**@GC. In each case, substrate is present in excess, and we observed that catalytic current increased linearly with concentration of substrate (Fig. 3), so the reactions were modelled as pseudo-first order in substrate.^16^Eqn (1) describes the anodic peak current density in the absence of substrate (*j*_p_). The catalytic current density (*j*_c_), for the case where catalyst (or mediator) is immobilized, in the presence of substrate, is described by eqn (2):^14a,b^

2 *j*_c_ = *nFk*_cat_*Γ*_1_[sub]

In eqn (2), *n* is the number of electrons passed (*n* = 2 for **5a**, *n* = 4 for **6a**), *k*_cat_ is the rate of reaction (M^–1^ s^–1^) and [sub] is concentration of substrate (M) (**5a** or **6a**). Other symbols were defined earlier. If eqn (2) is divided by eqn (1), the dependence on mediator surface coverage is eliminated and eqn (3) is obtained:^14a,b^
3
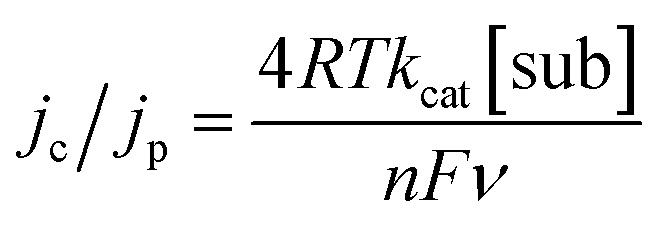



**Fig. 4:**
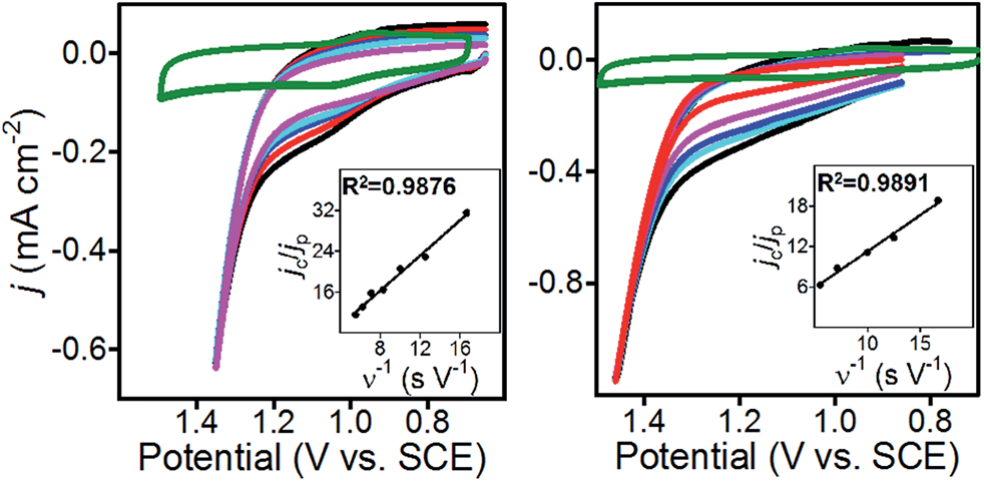
(Left) CV of **1**@GC (green); and with 80 mM **5a**, 200 mM 2,6-lutidine inset: plot of *j*_c_/*j*_p_ *vs.* inverse scan rate. (right) CV of **1**@GC (green); and with 80 mM **6a**, 200 mM 2,6-lutidine. Inset: plot of *j*_c_/*j*_p_ *vs.* inverse scan rate. All CVs recorded in 0.1 M Bu_4_NBF_4_ MeCN solution, at 180, 140, 100, 80, 60, 40 and 20 mV s^–1^.

CVs of **1**@GC were recorded in solution without substrate present, to obtain a value for *j*_p_, and in the presence of substrate at a series of increasing scan rates, to obtain values of *j*_c_ (Fig. 4). Using plots of *j*_c_/*j*_p_ *vs. ν* ^–1^, *k*_cat_ was calculated from eqn (3), as 460 M^–1^ s^–1^ for **5a**, and 575 M^–1^ s^–1^ for **6a** (Table 2). These numbers were obtained by measuring *j*_c_ at the potential where CPE experiments were performed, and *j*_p_ at the anodic peak potential (1.10 V). Using *k*_cat_ and the concentration of substrate in each case, we calculate that the TOF for oxidation of **5a** and **6a** are 37 and 46 s^–1^, respectively, using eqn (4). All parameters in eqn (4) were defined earlier in the text:

4 TOF = *k*_cat_ [sub]

**Table 2:** Rate constants and turnover frequencies determined from CV measurements^*a*^

	Substrate	*k*_cat_ (M^–1^ s^–1^)	TOF^*b*^ (s^–1^)
**1**@GC	*p*-AnOH, **5a**	460	37 ± 0.7
**1**	*p*-AnOH, **5a**	105	8.4 ± 1.4
TAI^*d*^	*p*-AnOH, **5a**	75	5.9
**1**@GC	BMMB, **6a**	575	46 ± 0.7
**1**	BMMB, **6a**	4.1	0.33 ± 0.03
TAI^*d*^	BMMB, **6a**	^*c*^	^*c*^

^*a*^Results are average of at least 3 trials.

^*b*^TOF calculated from *k*_cat_ using eqn (4) and the concentration of substrate in CV experiments (80 mM).

^*c*^Value not reported in literature and cannot be calculated from available information.

^*d*^Values for TAI taken from literature. Ref. 16.

We also determined *k*_cat_ for the oxidation of substrates by **1** as a homogeneous solution, so that comparison can be made to *k*_cat_ and TOF values determined for substrate oxidation by **1**@GC. For a catalytic reaction performed with homogeneous mediator, the relationship between *j*_c_/*j*_p_ and *k*_cat_ is given by eqn (5):^17a–c^
5
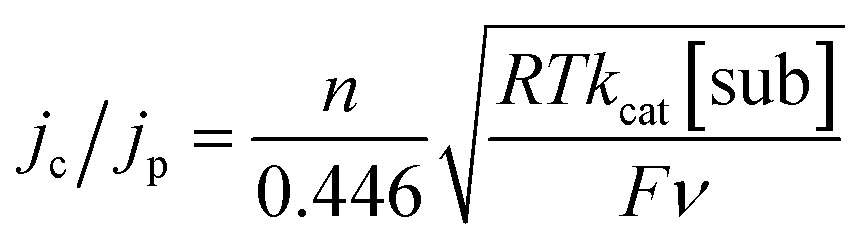



In eqn (5) all symbols have been defined earlier in the text. CVs of **1** with and without substrate were recorded at a series of scan rates to obtain *j*_p_ and *j*_c_ values, and then *k*_cat_ was determined to be 105 and 4.1 M^–1^ s^–1^ for the oxidation of **5a** and **6a**, respectively, using eqn (5) (Fig. S15†). Using eqn (4), the TOF is 8.4 and 0.33 s^–1^ for the oxidation of **5a** and **6a**, respectively (Table 2). Comparison of *k*_cat_ values for **1**@GC with **1** used as a homogeneous mediator for oxidation of **5a** and **6a** indicate a significant increase in reaction rate when **1** is immobilized, consistent with improved electron transfer from electrode to mediator when **1** is immobilized. Comparison of **1**@GC with previously reported rates of reaction for TAI reveal that *k*_cat_ is enhanced 5-fold using **1**@GC.^16^

## Conclusions

In summary, we have described a straightforward and reliable method to covalently bind the organic phenanthroimidazole (PIA) mediator to a GC electrode. Major advantages of this approach over previous work with homogeneous arylimidazole mediators, such as TAI and PIA, are enhanced stability and turnover, and the possibility of using an undivided electrochemical cell which is an advantage in large scale processes. We demonstrated that the enhanced stability of **1**@GC afforded by immobilization is manifested in observed TONs for oxidation of alcohol (**5a**) and ether (**6a**) that are 3 orders of magnitude greater than TONs previously observed when PIA is used as mediator in homogeneous solution. Oxidation is completely selective to afford the aldehyde and ester products, respectively. We believe that the longer life of the catalytic systems stems from stabilization of the intermediate, [**1**@GC]^+^˙, which cannot aggregate when adhered to a surface. Furthermore, we see an increase in reaction rates when **1** is immobilized on GC, compared to **1** in solution, which is most likely due to enhanced charge transfer.
