# Supplementary material for: High turnover in electro-oxidation of alcohols and ethers with a glassy carbon-supported phenanthroimidazole mediator
Source: Chem Sci. 2017 Jul 17;8(9):6493–8. doi: 10.1039/c7sc02482g (PMC5628575; doi:10.1039/c7sc02482g)
Supplement: Supplementary file 1 [file SC-008-C7SC02482G-s001.pdf]

Electronic Supplementary Material (ESI) for Chemical Science.
This journal is © The Royal Society of Chemistry 2017

Supporting Information to Accompany:

High Turnover in Electro-oxidation of Alcohol and Ether by a Carbon Electrode-Supported Phananthroimidazole Mediator

Bruce M. Johnson,[1] Robert Francke,[2*] R. Daniel Little,[3*] and Louise A. Berben[1*]

1. Department of Chemistry, University of California, Davis, CA 95616
2. Institut für Chemie, Universität Rostock, Albert-Einstein-Str. 3a, 18059 Rostock, Germany
3. Department of Chemistry and Biochemistry, University of California, Santa Barbara, CA 93106

email to: laberben@ucdavis.edu
robert.francke@uni-rostock.de
little@chem.ucsb.edu

Experimental Section

Preparation of Compounds. All manipulations with regard to mediator immobilization and electrode preparation were carried out using standard Schlenk or glove-box techniques under a dinitrogen atmosphere. Unless otherwise noted, solvents were deoxygenated and dried by thorough sparging with Argon (Praxair, 99.998%) gas followed by passage through an activated alumina column. Deuterated solvents were purchased from Cambridge Isotopes Laboratories, Inc., degassed and stored over activated 3 Å Molecular Sieve prior to use. All other reagents were purchased from commercial vendors and used without further purification. 2-Phenyl-1*H*-phenanthro[9,10-*d*]imidazole (precursor for compound **1**) and 2-(4-Methoxyphenyl)-1*H*-phenanthro[9,10-*d*]imidazole (precursor for compound **2**) were prepared as previously reported.[1]

2-Phenyl-1-propargylphenanthro[9,10-*d*]imidazole (1).

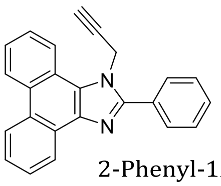

2-Phenyl-1*H*-phenanthro[9,10-*d*]imidazole (1.00 g, 3.40 mmol), K_2CO_3 (0.94 g, 6.80 mmol), propargyl bromide (80% in toluene, 0.76 mL, 1.01 g, 6.80 mmol) and 20 mL dry DMF were placed in a thick-walled glass tube sealed with a screw-on Teflon cap. The mixture was heated to 70 °C and stirred overnight. After cooling to room temperature, 10 mL of aq. ammonium hydroxide solution (10%) were added, followed by stirring for 30 min. The resulting suspension was then transferred into a separation funnel, together with dichloromethane (200 mL) and water (200 mL). After shaking, the organic layer was separated, washed with water (3 x 50 mL) and dried over sodium sulfate. The solvent is removed under reduced pressure and the crude product purified using column chromatography (toluene/ethyl acetate 95:5). Yield: 0.95 g (2.86 mmol, 84%). ^{1}H NMR (300 MHz, $CDCl_3$): δ(ppm) = 8.78 - 8.90 (m, 2H), 8.70 (d, *J* = 8.2 Hz, 1H), 8.61 (d, *J* = 8.0 Hz, 1H), 7.98 - 8.04 (m, 2H), 7.52-7.75 (m, 7H), 5.21 (d, *J* = 2.5 Hz, 2 H), 2.67 (t, *J* = 2.5 Hz, 1H). ^{13}C NMR (75 MHz, $CDCl_3$): δ(ppm) = 151.7, 136.7, 129.8, 129.7, 129.3, 129.0, 128.3, 127.6, 127.4, 127.1, 126.6, 126.4, 125.7, 125.4, 124.4, 123.6, 122.4, 121.9, 121.7, 79.1, 77.7, 37.7. Exact mass (ESI (+)): Calc. 333.1386 (M-H$^+$), Exp.: 333.1390. Elemental analysis: Calc. C 86.72, H 4.85, N 8.43; Exp.: C 86.45, H 4.87, N 8.30.

2-(4-Methoxyphenyl)-1-propargylphenanthro[9,10-*d*]imidazole (2).

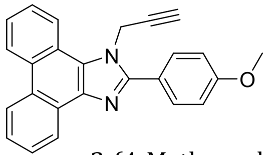

2-(4-Methoxyphenyl)-1*H*-phenanthro[9,10-*d*]imidazole (215 mg, 0.663 mmol), K_2CO_3 (183 mg, 1.326 mmol), propargyl bromide (80% in toluene, 0.148 g, 1.01 g, 0.995 mmol) and 8 mL dry DMF were placed in a thick-walled glass tube sealed with a screw-on Teflon cap. The mixture was heated to 70 °C and stirred overnight. After cooling to room temperature, 5 mL of aq. ammonium hydroxide solution (10%) were added, followed by stirring for 30 min. The resulting suspension was then transferred into a separation funnel, together with dichloromethane (50 mL) and water (50 mL). After shaking, the organic layer was separated, washed with water (3 x 20 mL) and dried over sodium sulfate. The solvent was removed under reduced pressure and the crude product purified using column chromatography (toluene/ethyl acetate 95:5). Yield: 205 mg (0.566 mmol, 85%). ^{1}H NMR (300 MHz, $CDCl_3$): δ(ppm) = 8.86 – 8.79 (m, 2H), 8.71 (d, *J* = 7.6 Hz, 1H), 8.61 (d, *J* = 6.9 Hz, 1H,) 7.94 (m, 2H), 7.67 (m, 4H), 7.12 (m, 2 H), 5.20 (d, *J* = 2.4 Hz, 2H), 3.92 (s, 3H), 2.66 (t, *J* = 2.4 Hz, 1H).^{13}C NMR (75 MHz, $CDCl_3$): δ(ppm) = 160.9, 152.3, 137.4, 131.1, 129.1, 128.1, 127.1, 127.1, 126.8, 126.7, 125.5, 124.9, 124.2, 123.0, 122.9, 122.6, 122.2, 121.1, 114.4, 78.7, 75.1, 55.4, 37.9. Exact mass (ESI (+)): Calc. 363.1492 (M-H$^+$), Exp.: 363.1495. Elemental analysis: Calc. C 82.85, H 5.01, N 7.73; Exp.: C 82.70, H 5.02, N 7.64.

2-(4-Hydroxyphenyl)-1*H*-phenanthro[9,10-*d*]imidazole.

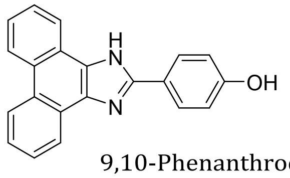

9,10-Phenanthroquinone (4.16 g, 20 mmol), ammonium acetate (15 g, 200 mmol) und 4-hydroxybenzaldehyde (2.44 g, 20 mmol) were dissolved in 50 mL DMSO and stirred at 95 °C for 1 h. After cooling to room temperature and filtration (for removal of solid impurities), 200 mL deionized water and 100 mL saturated K_2CO_3-solution were added slowly to the filtrate to precipitate the imidazol compound. The precipitate was filtered off, washed with water and small amounts of cold $CHCl_3$ and dried in vacuum. Yield: 5.45 g (17.56 mmol, 88%). ^{1}H NMR (300 MHz, DMSO-d_6): δ(ppm) = 13.21 (br. S., 1H), 9.92 (br. S., 1H), 8.83 (dd, *J*=8.8, 8.8 Hz, 2H), 8.58 (d, *J*=7.5 Hz, 1H), 8.53 (d, *J*=8.1 Hz, 1H), 8.16 (d, *J*=8.7 Hz, 2H), 7.73 (m, 2H), 7.54 - 7.66 (m, 2H), 6.99 (d, *J*=8.7 Hz, 2H). ^{13}C NMR (75 MHz, DMSO-d_6): δ(ppm) = 158.6, 149.7, 136.8, 127.8, 127.3, 127.2, 126.9, 126.9, 124.9, 124.8, 124.0, 123.6, 122.4, 121.8, 121.8, 121.7, 121.5, 115.6. Exact mass (ESI (+)): Calc. 311.1179 (M-H$^+$), Exp.: 311.1183.

2-(4-Propargyloxyphenyl)-1*H*-phenanthro[9,10-*d*]imidazole.

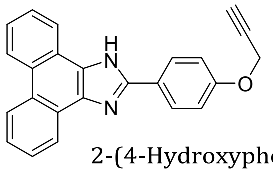

2-(4-Hydroxyphenyl)-1*H*-phenanthro[9,10-*d*]imidazole (500 mg, 1.61 mmol), K_2CO_3 (490 mg, 3.54 mmol), propargyl bromide (80% in toluene, 250 mg, 1.69 mmol) and 10 mL dry DMF were placed in a thick-walled glass tube sealed with a screw-on Teflon cap. The mixture was heated to 40 °C and stirred overnight. After cooling to room temperature, 5 mL of aq. ammonium hydroxide solution (10%) were added, followed by stirring for 30 min. The resulting suspension was then transferred into a separation funnel, together with dichloromethane (100 mL) and water (100 mL). After shaking, the organic layer was separated, washed with water (3 x 25 mL) and dried over sodium sulfate. The solvent is removed under reduced pressure and the crude product purified using column chromatography (toluene/ethyl acetate 4:1). Yield: 0.366 g (1.05 mmol, 65%). ^{1}H NMR (250 MHz, DMSO-d_6): δ(ppm) = 13.30 (s, 1 H), 8.70-8.90 (m, 2 H), 8.57 (d, *J*=7.7 Hz, 1H), 8.51 (d, *J*=7.9 Hz, 1 H), 8.25 (d, *J*=9.0 Hz, 2 H), 7.65 - 7.78 (m, 2H), 7.55 - 7.65 (m, 2H), 7.21 (d, *J*=8.8 Hz, 2H), 4.91 (d, *J*=2.4 Hz, 2H), 3.61 (t, *J*=2.4 Hz, 1H). ^{13}C NMR (75 MHz, $CDCl_3$): δ(ppm) = 158.0, 149.1, 127.6, 127.4, 127.4, 127.0, 126.9, 125.2, 124.9, 124.0, 123.7, 123.6, 122.4, 121.8, 115.2, 79.0, 78.4, 55.5. Exact mass (ESI (+)): Calc. 349.1335 (M-H$^+$), Exp.: 349.1338.

2-(4-Propargyloxyphenyl)-1methylphenanthro[9,10-*d*]imidazole (3).

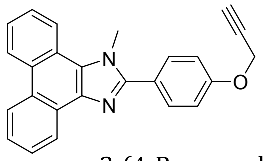

2-(4-Propargyloxyphenyl)-1*H*-phenanthro[9,10-*d*]imidazole (250 mg, 0.718 mmol), K_2CO_3 (198 mg, 1.435 mmol), iodomethane (0.3 mL, 132 mg, 0.927 mmol) and DMF (10 mL) were placed in a thick-walled glass tube sealed with a screw-on Teflon cap. The mixture was stirred at room temperature overnight. After completed reaction, 5 mL of aq. ammonium hydroxide solution (10%) were added, followed by stirring for 30 min. The resulting suspension was then transferred into a separation funnel, together with dichloromethane (50 mL) and water (50 mL). After shaking, the organic layer was separated, washed with water (3 x 20 mL) and dried over sodium sulfate. The solvent was removed under reduced pressure and the crude product purified using column chromatography (toluene/ethyl acetate 95:5). Yield: 215 mg (0.593 mmol, 83%). ^{1}H NMR (300 MHz, $CDCl_3$): δ(ppm) = 8.77 - 8.86 (m, 2H), 8.77 - 8.86 (m, 2H), 8.67 - 8.73 (m, 1H), 8.41 - 8.46 (m, 1H), 7.58 - 7.80 (m, 6H), 7.12 - 7.20 (m, 2H), 4.79 (d, *J*=2.5 Hz, 2H), 4.26 (s, 3H), 2.58 (t, *J*=2.5 Hz, 1H). ^{13}C NMR (75 MHz, $CDCl_3$): δ(ppm) = 157.4,

151.3, 130.2, 128.0, 126.9, 126.4, 126.3, 126.1, 125.5, 124.3, 123.6, 123.3, 122.6, 122.0, 121.5, 119.5, 114.1, 77.1, 74.9, 54.9, 35.04. Exact mass (ESI (+)): Calc. 363.1492 (M-H+), Exp.: 363.1496. Elemental analysis: Calc. C 82.85, H 5.01, N 7.73, Exp.: C 82.64, H 5.09, N 7.60.

2-(3-Hydroxyphenyl)-1H-phenanthro[9,10-*d*]imidazole.

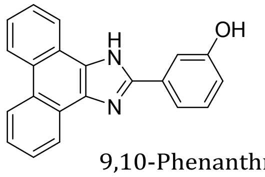

9,10-Phenanthroquinone (4.16 g, 20 mmol), ammonium acetate (15 g, 200 mmol) und 3-hydroxybenzaldehyde (2.44 g, 20 mmol) were dissolved in 50 mL DMSO and stirred at 95 °C for 1 h. After cooling to room temperature and filtration (for removal of solid impurities), 200 mL deionized water and 100 mL saturated K_2CO_3-solution were added slowly to the filtrate to precipitate the imidazol intermediate. The precipitate is filtered off, washed with water and small amounts of cold CH_2Cl_2 and dried in vacuum. Yield: 5.21 g (16.79 mmol, 84%). ^{1}H NMR (300 MHz, DMSO-d_6): δ(ppm) = 13.34 (br. s., 1H), 9.80 (br. s., 1H), 8.84 (d, *J*=8.1 Hz, 2H), 8.59 (d, *J*=6.8 Hz, 2H), 7.59 - 7.81 (m, 6H), 7.35 – 7.45 (m. 1H), 6.90 - 6.95 (m, 1H). ^{13}C NMR (75 MHz, DMSO-d_6): δ(ppm) = 157.8, 149.2, 131.6, 129.9, 127.5, 127.0, 125.1, 123.8, 121.9, 116.9, 116.3, 113.0. Exact mass (ESI (+)): Calc. 311.1179 (M-H+), Exp.: 311.1172.

2-(3-Propargyloxyphenyl)-1*H*-phenanthro[9,10-*d*]imidazole.

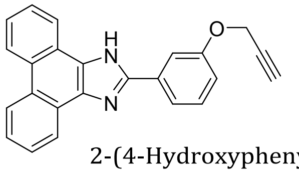

2-(4-Hydroxyphenyl)-1*H*-phenanthro[9,10-*d*]imidazole (700 mg, 2.256 mmol), K_2CO_3 (680 mg, 4.962 mmol), propargyl bromide (80% in toluene, 353 mg, 2.369 mmol) and 14 mL dry DMF were placed in a thick-walled glass tube sealed with a screw-on Teflon cap. The mixture was heated to 40 °C and stirred overnight. After cooling to room temperature, 5 mL of aq. ammonium hydroxide solution (10%) were added, followed by stirring for 30 min. The resulting suspension was then transferred into a separation funnel, together with dichloromethane (100 mL) and water (100 mL). After shaking, the organic layer was separated, washed with water (3 x 25 mL) and dried over sodium sulfate. The solvent was removed under reduced pressure and the crude product purified using column chromatography (toluene/ethyl acetate 95:5). Yield: 0.472 g (1.355 mmol, 60%). ^{1}H NMR (300 MHz, $CDCl_3$): δ(ppm) =. 13.40 (s, 1 H), 8.80 – 8.87 (m, 2 H), 8.60 – 8.51 (m, 2 H), 7.90 - 7.95 (m, 2 H), 7.49 - 7.77 (m, 5 H), 7.10 - 7.15 (m, 1 H), 4.94 (d, *J*=2.5 Hz, 2 H), 3.62 (t, *J*=2.4 Hz, 1 H). ^{13}C NMR (75 MHz, $CDCl_3$): δ(ppm) = 157.6, 148.6, 136.8, 131.6, 130.0, 127.6, 127.6, 127.5, 127.1, 127.0, 126.8, 125.3, 125.1, 124.0, 123.6, 122.3, 121.9, 121.8, 119.1, 115.4, 112.6, 79.1, 78.4, 55.6. Exact mass (ESI (+)): Calc. 349.1335 (M-H+), Exp.: 349.1339.

2-(3-Propargyloxyphenyl)-1methylphenanthro[9,10-*d*]imidazole (4).

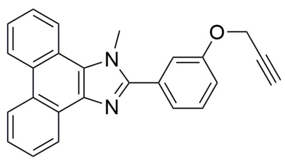

2-(3-Propargyloxyphenyl)-1*H*-phenanthro[9,10-*d*]imidazole (250 mg, 0.718 mmol), K_2CO_3 (198 mg, 1.435 mmol), iodomethane (0.3 mL, 132 mg, 0.927 mmol) and DMF (10 mL) were placed in a thick-walled glass tube sealed with a screw-on Teflon cap. The mixture was stirred at room temperature overnight. After completed reaction, 5 mL of aq. ammonium hydroxide solution (10%) were added, followed by stirring for 30 min. The resulting suspension was then transferred into a separation funnel, together with dichloromethane (50 mL) and water (50 mL). After shaking, the organic layer was separated, washed with water (3 x 20 mL) and dried over

sodium sulfate. The solvent was removed under reduced pressure and the crude product purified using column chromatography (toluene/ethyl acetate 95:5). Yield: 234 mg (0.646 mmol, 90%). ^{1}H NMR (300 MHz, $CDCl_3$): δ(ppm) = 8.76 - 8.84 (m, 2H), 8.68 (d, *J*=7.7 Hz, 1H), 8.35 - 8.43 (m, 1H), 7.56 - 7.76 (m, 4H), 7.39 - 7.54 (m, 3H), 7.11 - 7.19 (m, 1H), 4.82 (d, *J*=2.4 Hz, 2H), 4.24 (s, 3H), 2.58 (t, *J*=2.4 Hz, 1H). ^{13}C NMR (75 MHz, $CDCl_3$): δ(ppm) =. 157.7, 152.1, 137.4, 131.7, 129.8, 129.1, 128.2, 128.0, 127.6, 127.2, 126.5, 125.4, 124.8, 124.3, 123.6, 123.1, 123.0, 122.5, 120.6, 116.3, 116.1, 78.4, 75.8, 56.0, 36.0. Exact mass (ESI (+)): Calc. 363.1492 (M-H^+), Exp.: 363.1495. Elemental analysis: Calc. C 82.85, H 5.01, N 7.73, Exp.: C 82.96, H 5.17, N 7.49.

^{1}H NMR Measurements. ^{1}H-NMR spectra were recorded at ambient temperature using a Varian 600 MHz spectrometer (for reaction monitoring) or a Bruker AVANCE 300 MHz or 250 MHz spectrometer (synthesis of the precursor of the mediator). Chemical shifts are reported in parts per million and referenced to the signal of the residual non- or partially deuterated solvent.[2]

Preparation of Glassy Carbon (GC) for Immobilization. A 1 cm^2 GC plate (120 mg) was submerged in 5 mL H_2O and hydrazine (35 wt% in water, 0.235 mL) and ammonium hydroxide (28 wt% in water, 1.62 mL) were added. The suspension was heated to 95° C for 1 hour, then the solution was removed via cannula transfer and the plate and flask were dried under vacuum at 100° C. A solution of NaN_3 (0.02 g, 0.31 mmol) and ICl (0.01 g, 0.08 mmol) in 10 mL of hexanes was added to the flask and the solution mixture was stirred for 1 hour. The GC plate was then washed and sonicated in MeCN for 5 minutes.

Modification of GC with Mediators. Azide-terminated GC plates were modified with ethynyl-substituted **1-4** according to literature procedures.[3] It was noted, however, that the click reaction would not proceed in the presence of water. Consequently, dry DMSO was used and the reaction was carried out in flame dried glassware.

Electrochemical Measurements. Cyclic voltammograms were recorded under a dinitrogen (Praxair, 99.998%) atmosphere using a CH Instruments Electrochemical Analyzer Model 620D or 1100, a 1 cm^2 GC (Tokai Carbon) modified with the mediator of choice as the working electrode, a platinum wire auxiliary electrode, and a $Ag/AgNO_3$ non-aqueous reference electrode with a Vycor tip. Reported potentials are all referenced to the SCE couple, and were determined using ferrocene as an external standard where $E_{1/2}$ ferrocene/ferrocenium is +0.400 V vs. SCE in acetonitrile.[4] Bu_4NBF_4 was synthesized from the reaction of Bu_4NBr and HBF_4 in aqueous solution. The subsequent white powder was collected and rinsed with water several times to remove excess HBF_4. The product was recrystallized from 4:1 ethyl acetate:hexanes and dried under vacuum at 70 °C for 48 hours before use.[5] Non-aqueous electrolyte solutions were stored over 3 Å molecular sieves which had been activated by heating under vacuum at 200°C for at least 72 hours. Background corrected peak currents were obtained by subtracting the baseline currents before the onset of the peak from the peak currents. The background subtracted anodic peak current was then compared to the background subtracted cathodic peak current and a ratio of the two was obtained (i_{pa}/i_{pc}).

Controlled Potential Electrolysis (CPE). CPE experiments were performed in a custom designed gas-tight glass cell under 1 atm of static dinitrogen (Praxair, 99.998%). Solutions were sparged with N_2 prior to the commencement of the experiment. The counter electrode compartment was separated from the working electrode compartment by a glass frit of medium porosity. In a typical experiment, 20 mL of electrolyte solution were used in the working electrode compartment and 25 mL of electrolyte were used in the counter electrode compartment. A 1 cm^2 GC plate (Tokai Carbon) modified with the mediator of choice was used as the working electrode. The auxiliary electrode was a coiled Pt wire (BASi). In between CPE experiments, the cell was sonicated in 5% v/v nitric acid for 10 min, rinsed, sonicated in methanol for 10 min, rinsed, and sonicated in water for 10 min. The cell was then dried in an oven at 150 °C for a minimum of 2 hours.

CPE experiments for the oxidation of **5a** were also conducted in an undivided cell. **5a** (0.5 mmol, 0.062 mL) and 2,6-lutidine (2.5 mmol, 0.290 mL) were added to Bu_4NBF_4 MeCN electrolyte solution (10 mL) in a 4-necked round bottom flask. Experiments were performed under 1 atm of active N_2 using the same electrodes as described above for divided cell experiments. In between experiments, the round bottom flask was submerged in a base bath (saturated EtOH/KOH) for a minimum of 2 hours before being rinsed with 1.0 M HCl solution and distilled water. The flask was dried in an oven at 150 °C for a minimum of 2 hours.

Quantification of *p*-anisaldehyde (*p*-AnAld, **5b**) was performed using ^{1}H NMR spectroscopy. An internal standard of a known amount of dimethylformamide, as a dilute solution in 100% C_6D_6, was prepared and sealed in a glass capillary tube. 500 μL of the CPE solution were injected into an NMR tube with the internal standard

capillary. The integration of the 1-H resonance at 7.63 ppm for DMF,[2] was used to quantify **5b** produced (10.68 ppm). A similar method was used to quantify the amount of benzyl 4-methoxybenzoate (**6b**). 2-Bromobenzaldehyde of known concentration in 100% C_6D_6 was used as an internal standard. The integration of the 1-H resonance at 10.2 ppm for 2-bromobenzaldehyde was used to quantify **6b** produced (6.05 ppm).

Calculation S1. Theoretical Surface Coverage of Fc@GC. Assuming Fc is a rectangle that is approximately 3.00 Å × 2.08 Å lying flat on the GC surface, the maximum theoretical surface coverage for a monolayer of Fc on a 1 cm^2 GC plate would be 1.60×10^{15} molecules/cm^2 or 2.66×10^{-9} mol/cm^2.

$$\frac{1\ molecule}{(2.08 \times 10^{-8}\ cm)(3.00 \times 10^{-8}\ cm)} = 1.60 \times 10^{15}\ \text{molecules}\ cm^{-2} = 2.66 \times 10^{-9}\ \text{mol}\ cm^{-2}$$

Calculation S2. Determination of distance between molecules of 1@GC. The dimensions of **1** were calculated from typical bond lengths and atomic radii. We assume the GC electrode has an exact surface area ($SA_{elec.}$) of 1 cm^2 (1×10^{16} Å^2). Each molecule is evenly spaced from one another and **1** is represented as a rectangle with an area (A_1) of 39.9 Å^2 (11.75 Å long × 3.4 Å thick) standing on its side on the GC surface (see graphic below). The surface coverage (Γ) is 2.17×10^{14} molecules of **1** on the surface.

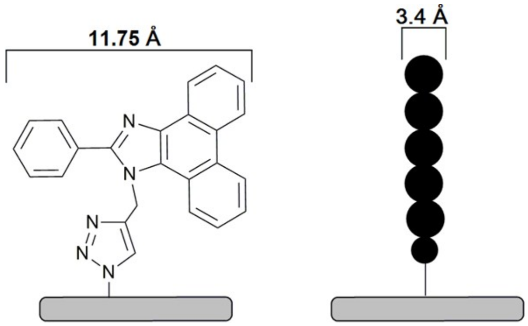

$$\text{Average Spacing} = \sqrt{\frac{SA_{elec.} - (A_1 \times \Gamma)}{\Gamma}}$$

$$\text{Average Spacing} = \sqrt{\frac{1 \times 10^{16}\ \text{Å}^2 - (39.9\ \text{Å}^2 \times 2.17 \times 10^{14})}{2.17 \times 10^{14}\ molecules}} = 2.50\ \text{Å}$$

Calculation S3. Calculation of TON. This describes moles of product produced per mole of catalyst and is based on mediator surface coverage. In a sample reaction for the oxidation of BMMB, **6a**, 0.00540 mmol of **6b** were produced in 10 hours at a modified electrode with **1** having surface coverage of 4.38×10^{-10} mol cm^{-2}. The GC electrode surface area ($SA_{elec.}$) was 1 cm^2.

$$\text{TON} = \frac{mol\ of\ oxidized\ product}{(1\ surface\ coverage)(SA_{elec.})}$$

$$\text{TON} = \frac{5.40 \times 10^{-6}\ mol\ product}{(4.38 \times 10^{-10}\ mol\ cm^{-2})(1\ cm^2)} = 12{,}300$$

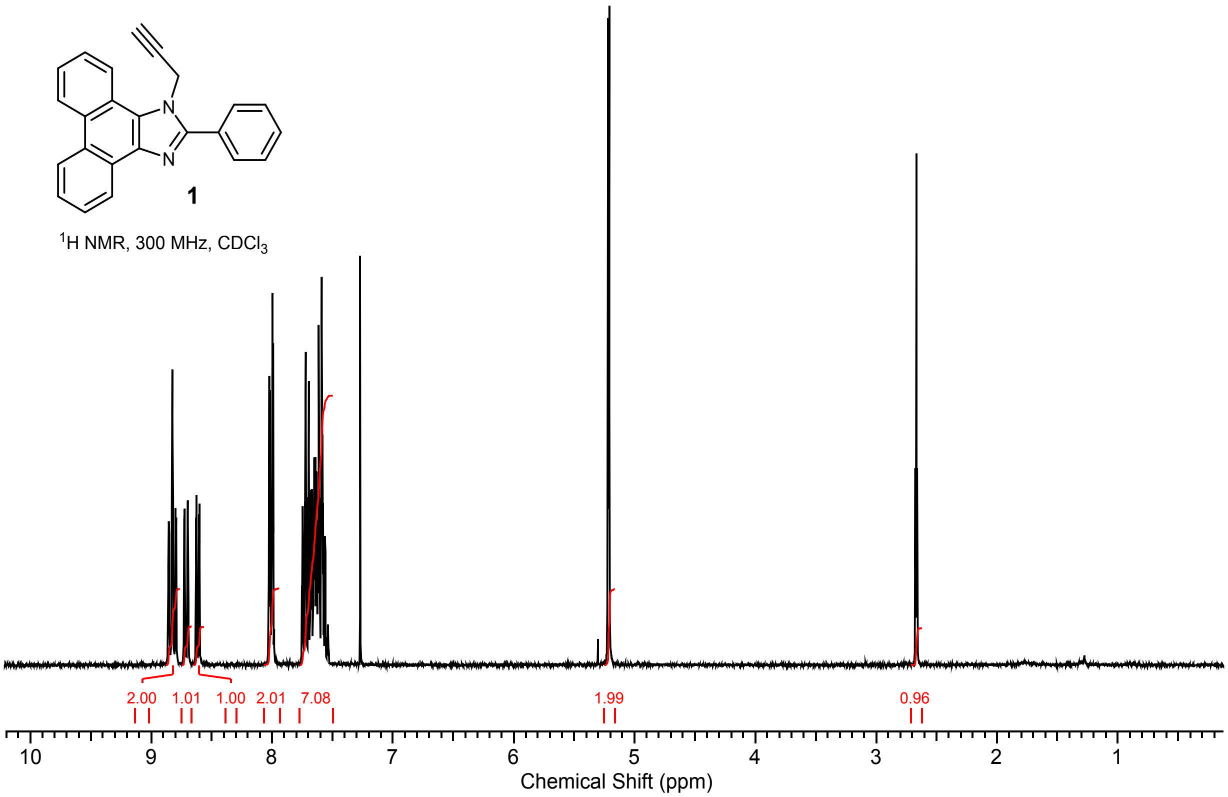

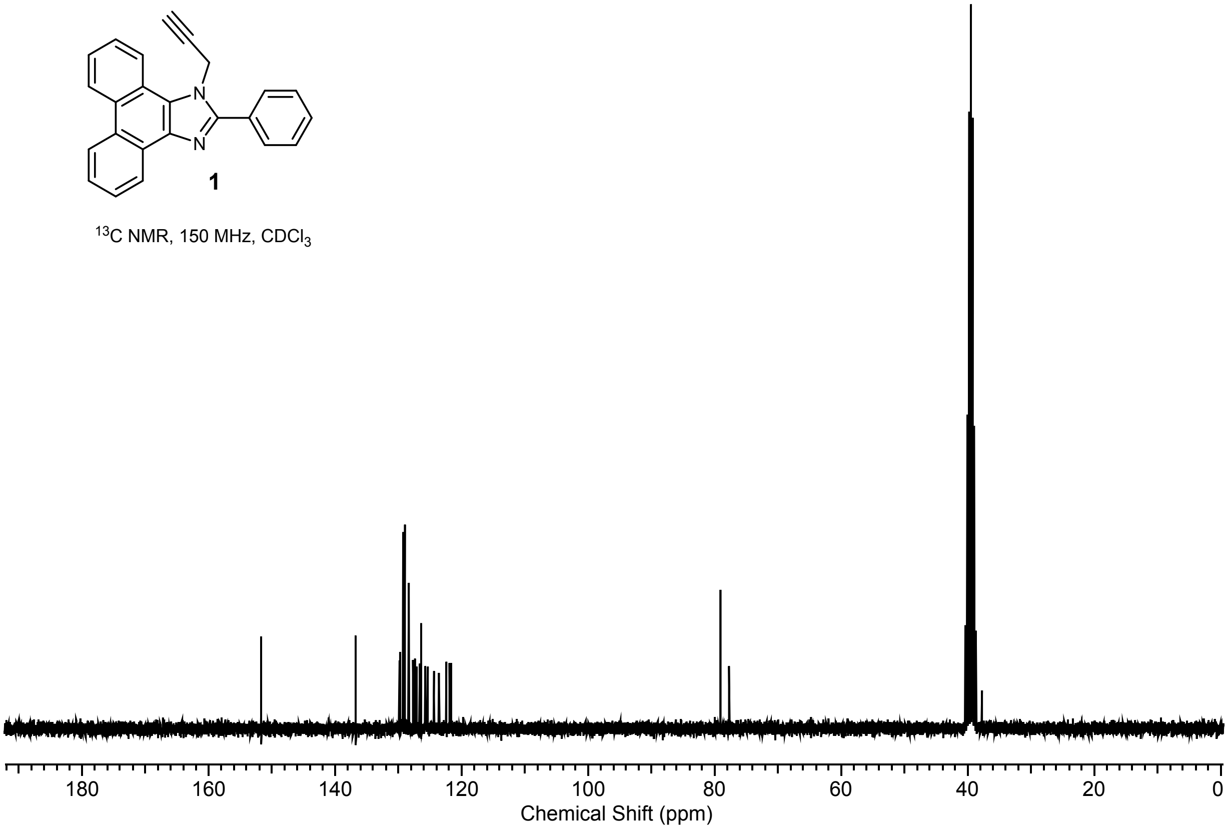

Figure S1. ^{1}H NMR (top) and ^{13}C NMR (bottom) of **1**.

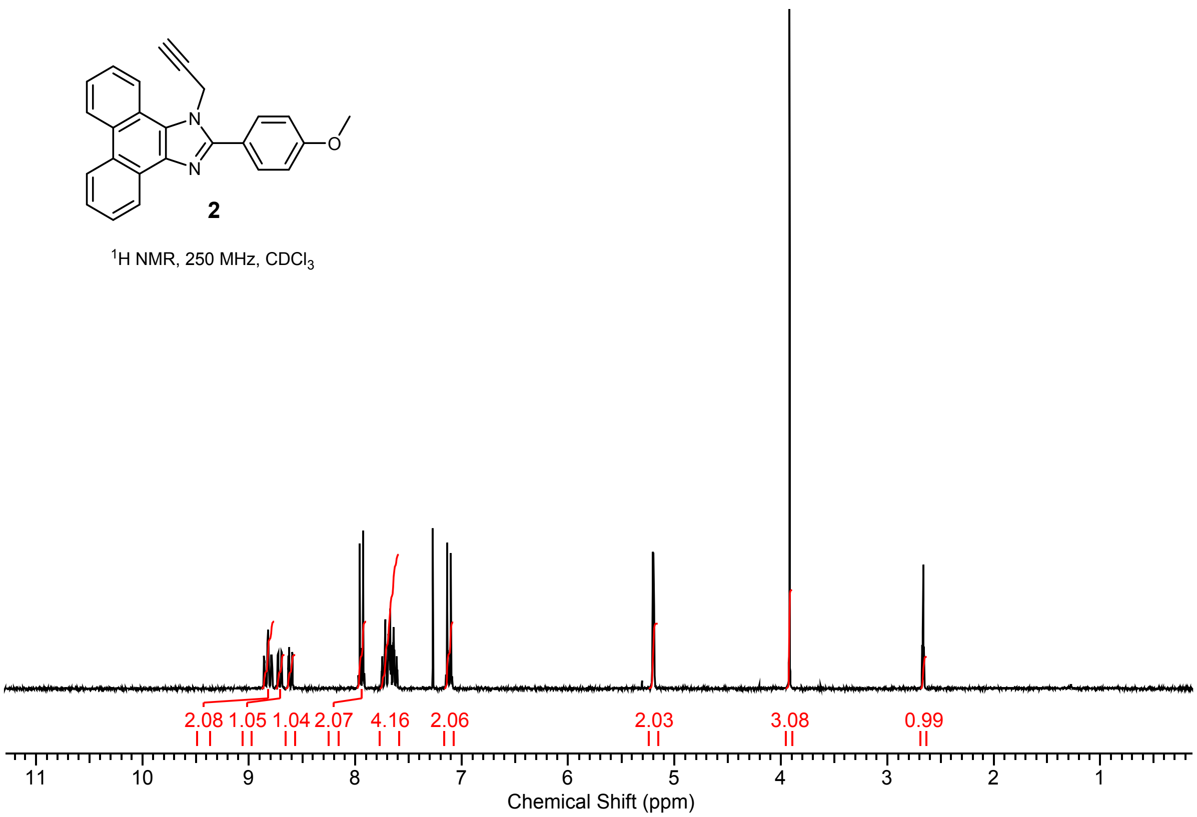

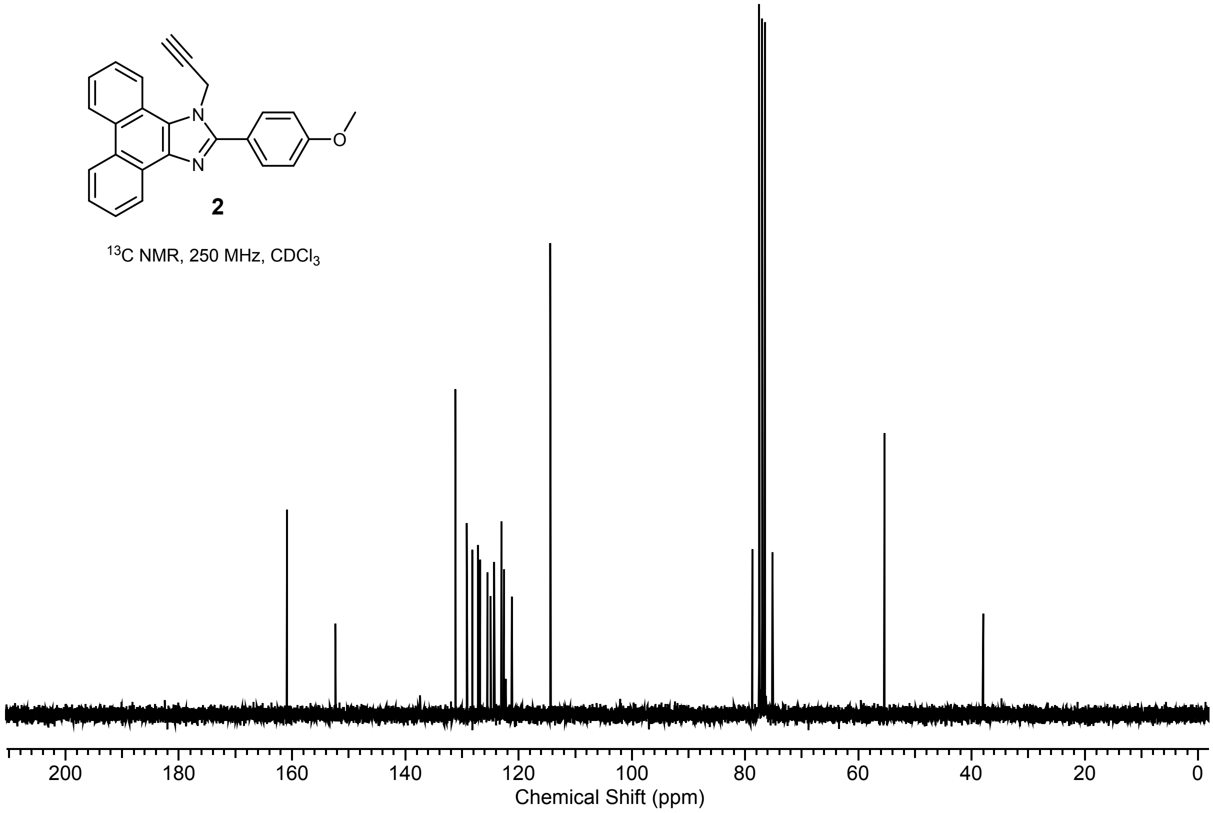

Figure S2. ^{1}H NMR (top) and ^{13}C NMR (bottom) of **2**.

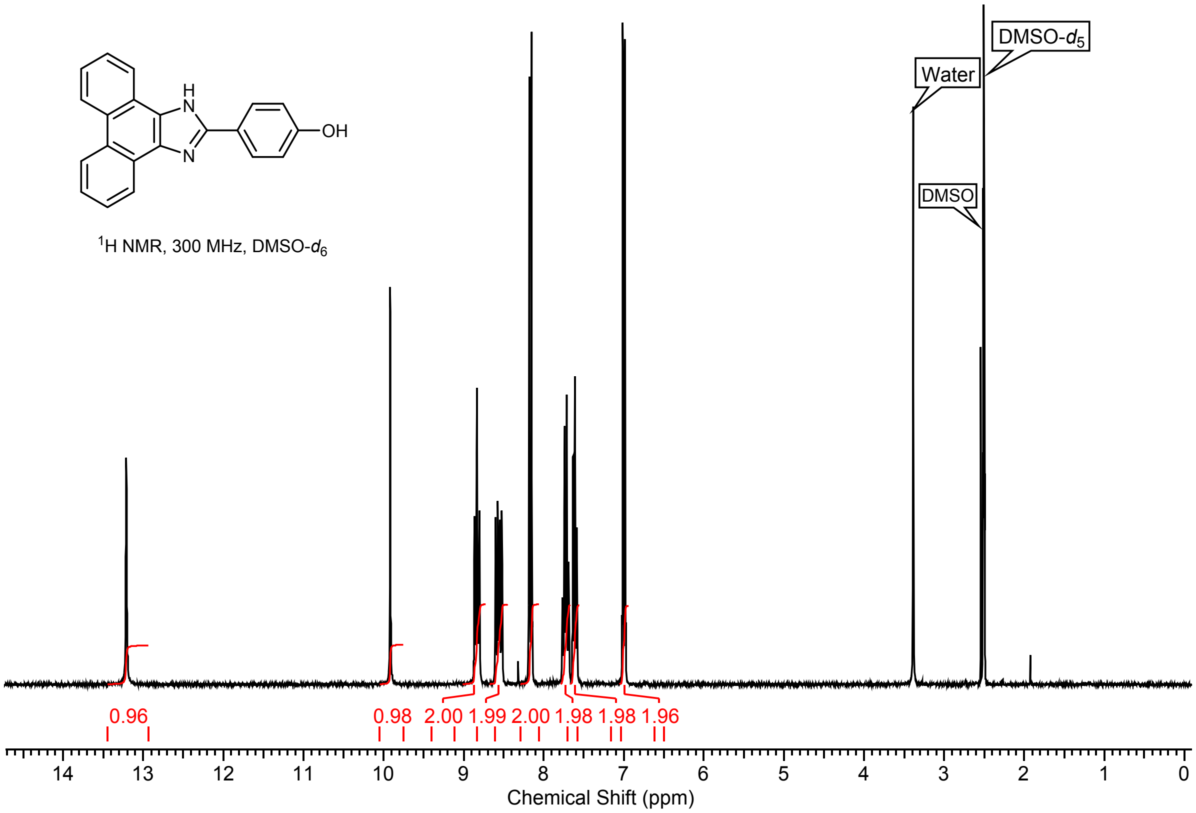

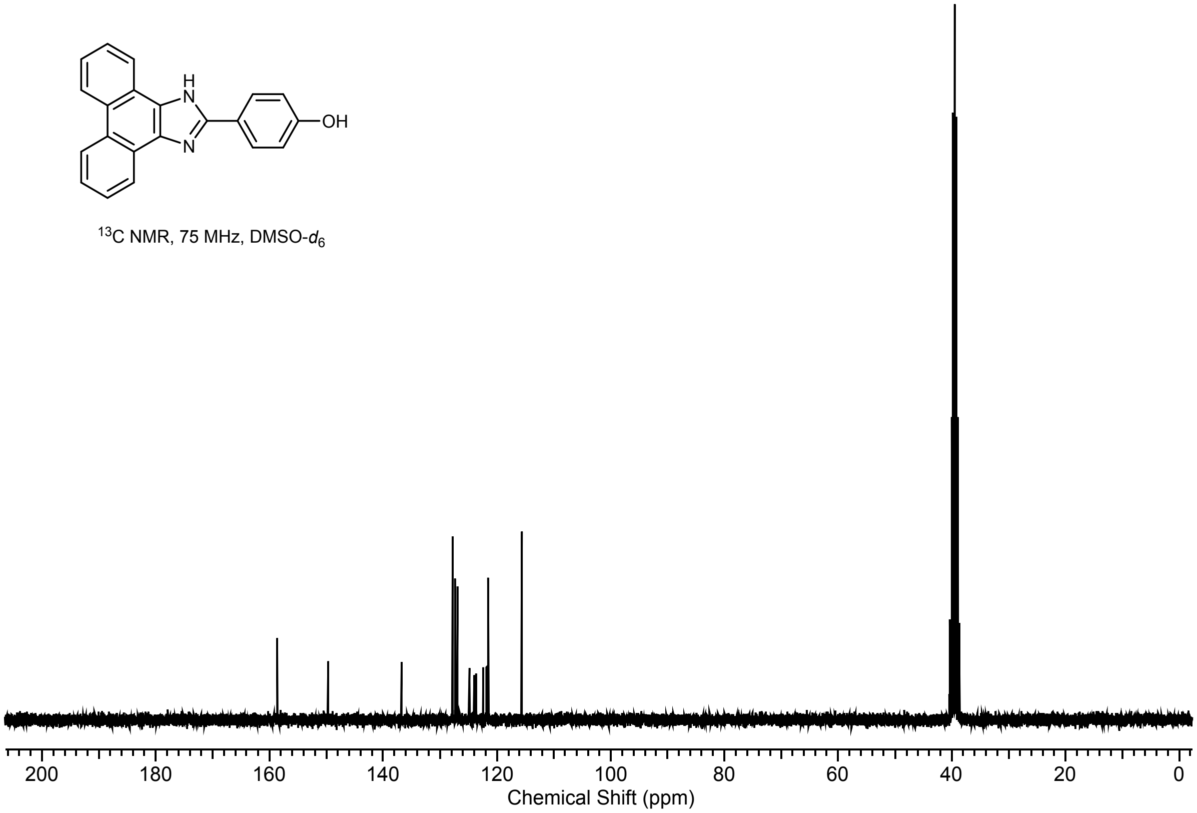

Figure S3. ^{1}H NMR (top) and ^{13}C NMR (bottom) of 2-(4-Hydroxyphenyl)-1*H*-phenanthro[9,10-*d*]imidazole.

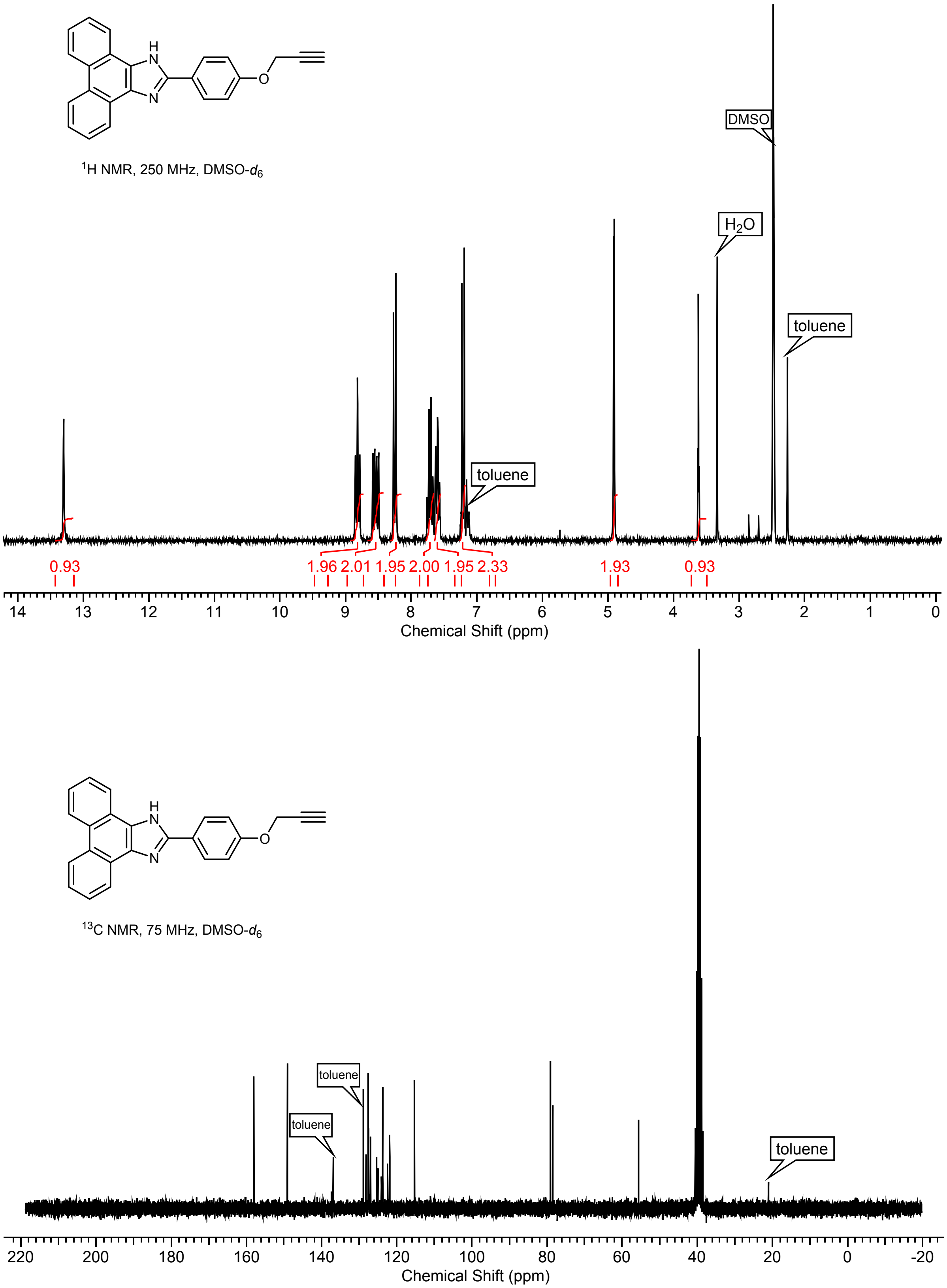

Figure S4. ^{1}H NMR (top) and ^{13}C NMR (bottom) of 2-(4-Propargyloxyphenyl)-1*H*-phenanthro[9,10-*d*]imidazole.

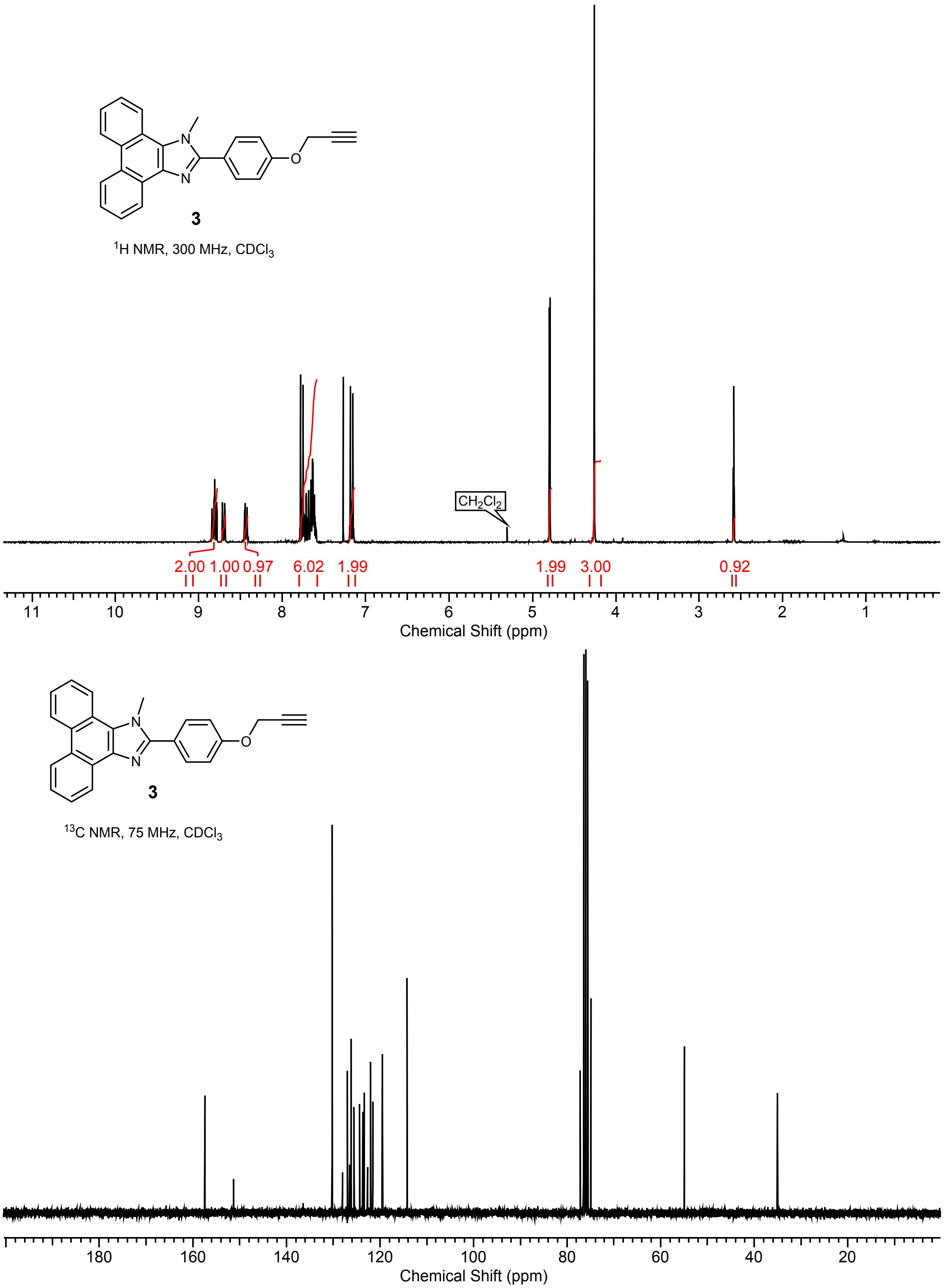

Figure S5. ^{1}H NMR (top) and ^{13}C NMR (bottom) of **3**.

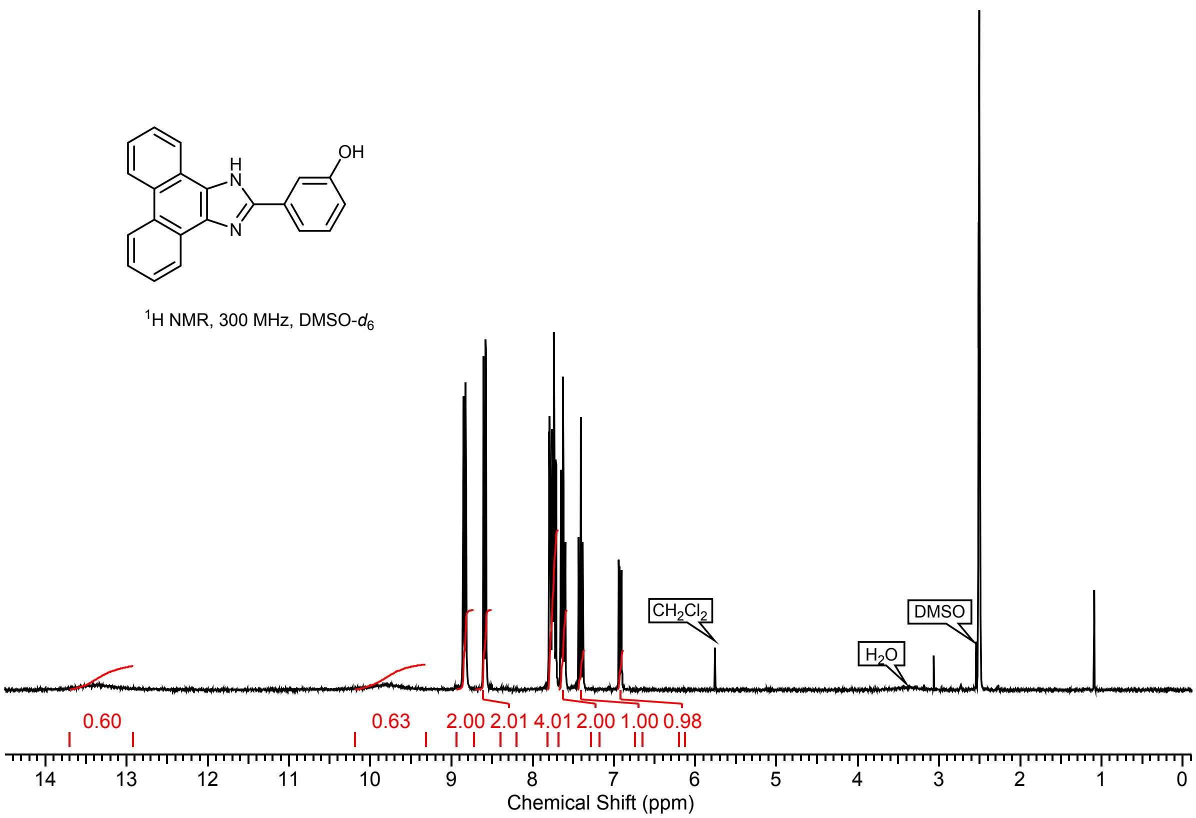

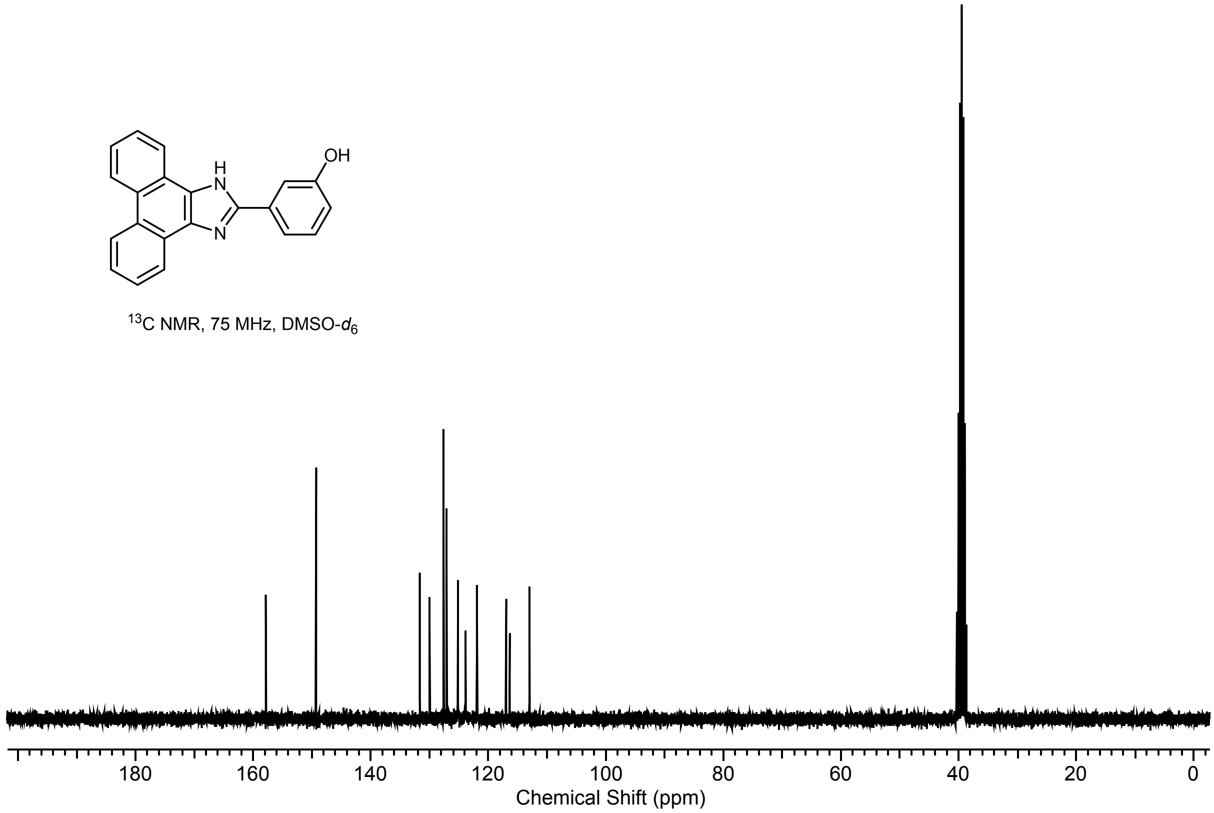

Figure S6. ^{1}H NMR (top) and ^{13}C NMR (bottom) of 2-(3-Hydroxyphenyl)-1*H*-phenanthro[9,10-*d*]imidazole.

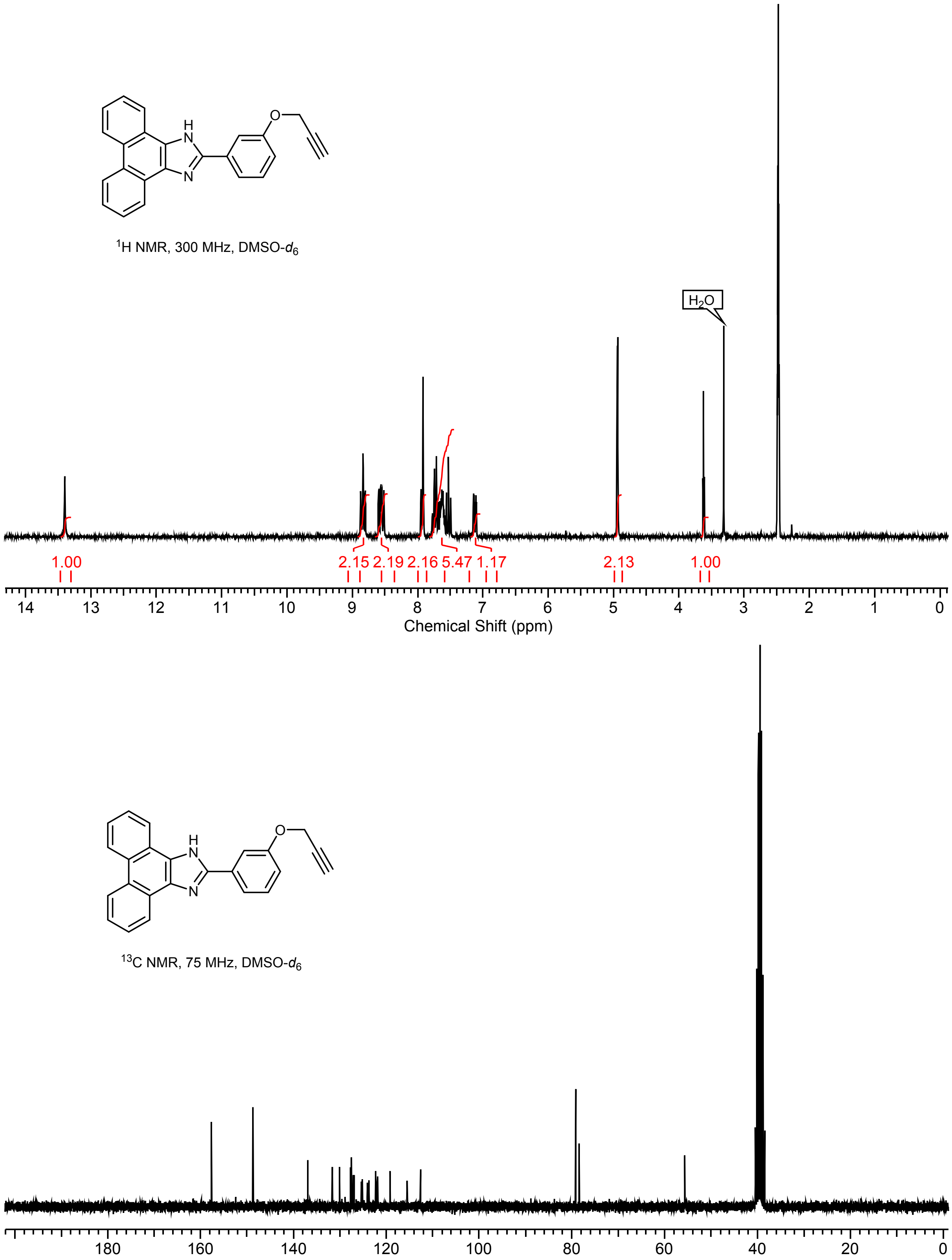

Figure S7. ^{1}H NMR (top) and ^{13}C NMR (bottom) of 2-(3-Propargylphenyl)-1*H*-phenanthro[9,10-*d*]imidazole.

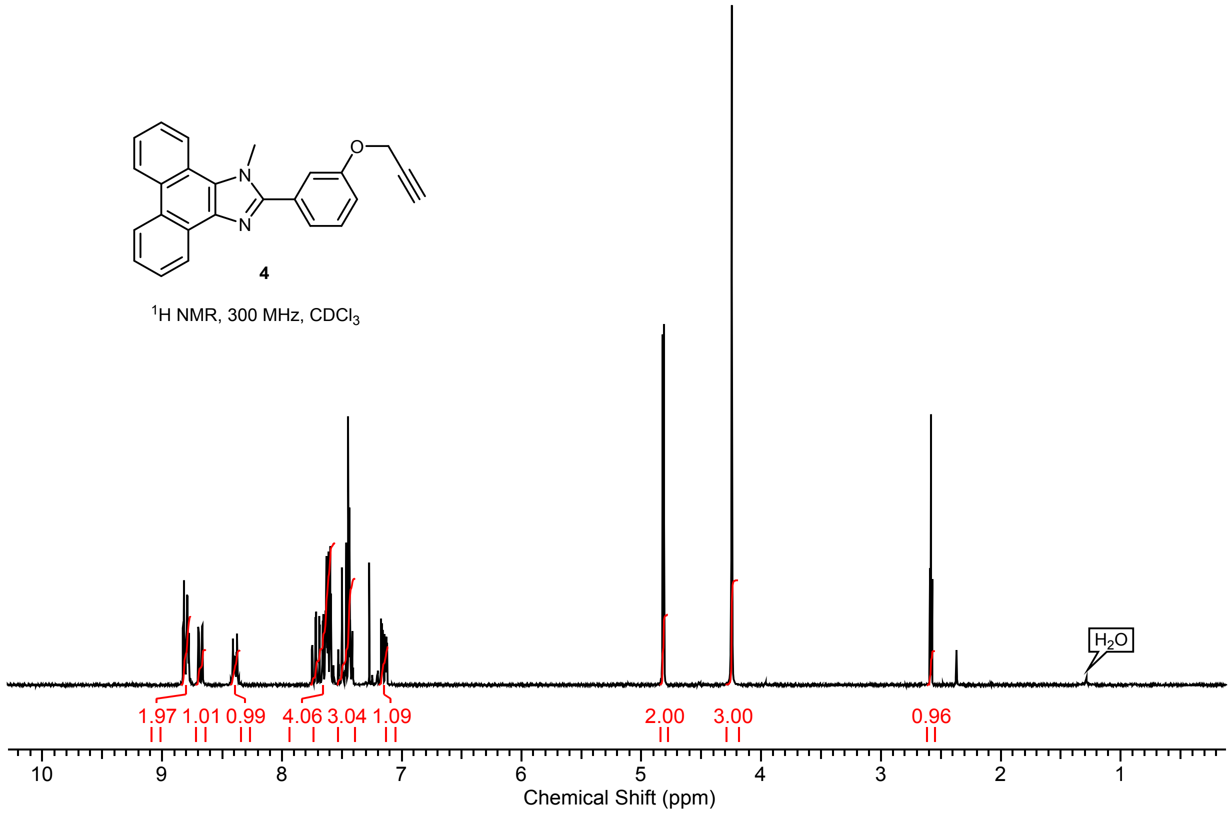

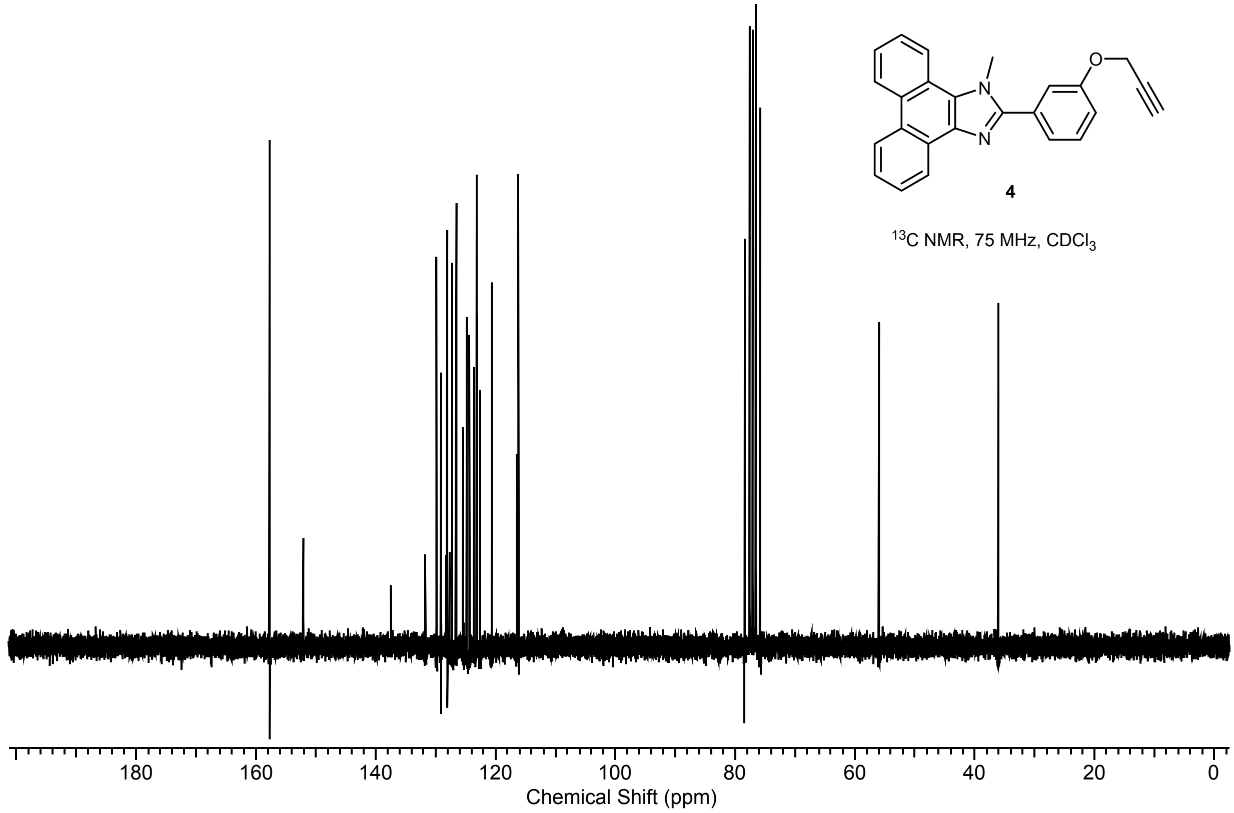

Figure S8. ^{1}H NMR (top) and ^{13}C NMR (bottom) of **4**.

Table S1. Surface attachment information for materials immobilized on GC.

Substrate	$E_{1/2}$ (V vs. SCE)	Surface Coverage (mol. cm^{-2})
Fc@GC[a]	0.29	3.93×10^{-10}
Fc@GC[6a]	0.38	6.64×10^{-10}
Fc@GC[6b]	0.38	3.32×10^{-11}
Fc@GC[6c]	0.30	8.10×10^{-10}
**1**@GC[a]	1.00	5.47×10^{-10}
**2**@GC[a]	1.10	8.32×10^{-10}
**3**@GC[a]	1.04	8.76×10^{-10}
**4**@GC[a]	1.05	3.31×10^{-10}

a) Experimental data from the current work.

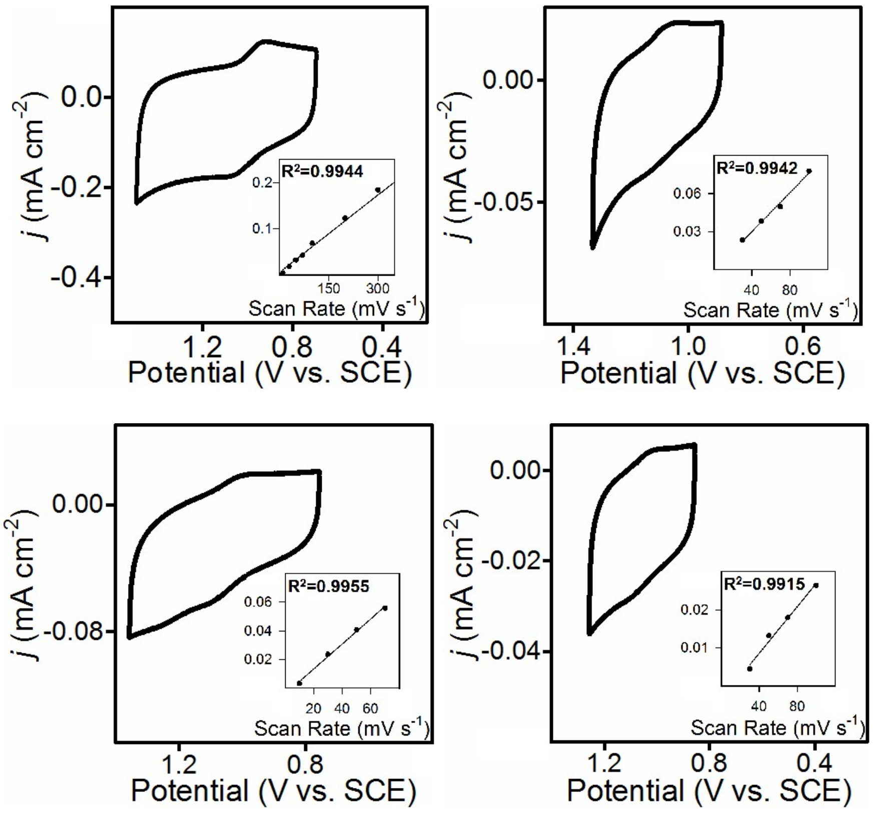

Figure S9. CVs of (top, left) **1**@GC, (top, right) **2**@GC, (bottom, left) **3**@GC and (bottom, right) **4**@GC. (Inlays) Linear peak current density vs. scan rate plots. All CVs were recorded in 0.1 M Bu_4NBF_4 MeCN electrolyte solution.

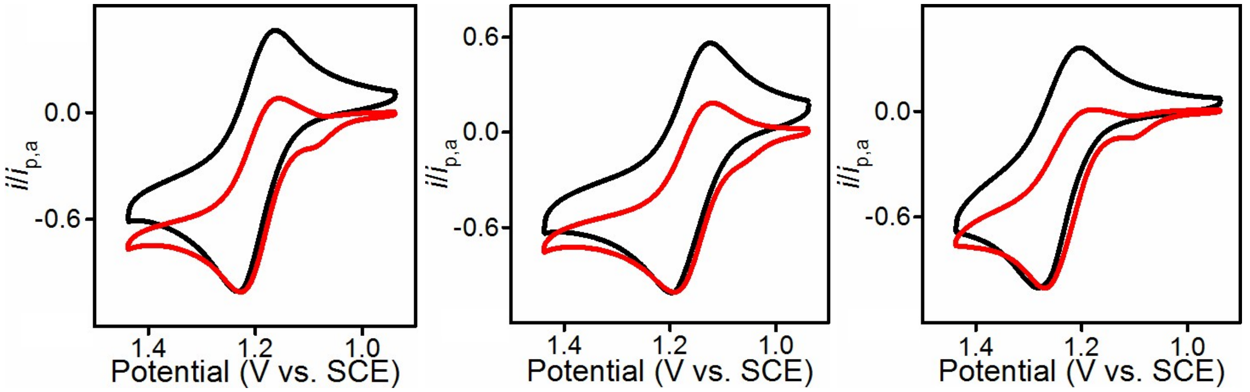

Figure S10. CVs of 1 mM (left) **2**, (middle) **3** and (right) **4** at (black) 100 mV/s and (red) 10 mV/s. Each scan has been normalized to the anodic peak current, $i_{p,a}$. All CVs were recorded in 0.1 M Bu_4NBF_4 MeCN electrolyte solution.

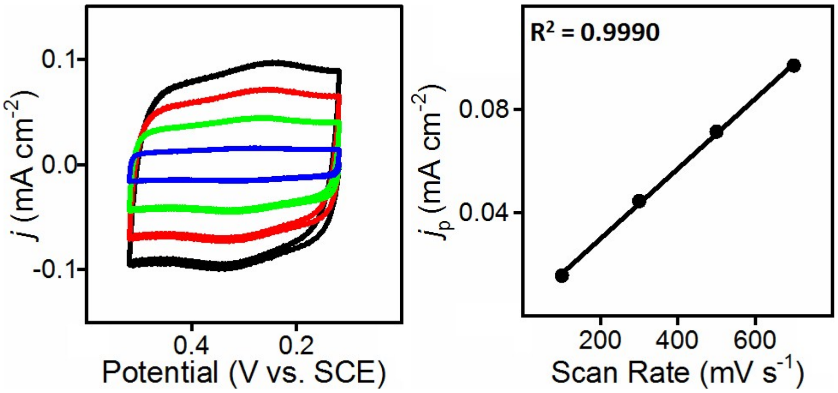

Figure S11. (Left) CVs of Fc@GC at 100, 300, 500 and 700 mV/s. All CVs were recorded in 0.1 M $NaHCO_3$ aqueous electrolyte solution. (Right) Plot of peak current vs. scan rate for Fc@GC. Surface coverage was calculated to be 3.93×10^{-10} mol cm^{-2}.

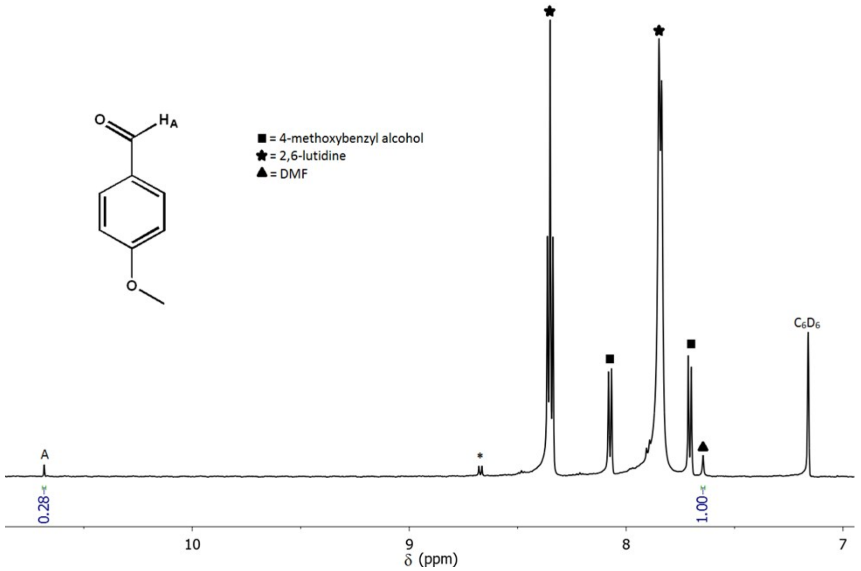

Figure S12. ^{1}H NMR spectrum of the electrolyte solution from a CPE experiments for oxidation of *p*-AnOH by **1**@GC. Internal standard of DMF/C_6D_6 added as capillary to NMR tube. The CPE experiment was run for 5.5 hours at 1.37 V vs. SCE using a 1 cm^2 GC plate modified with **1** as the working electrode. The 0.1 M Bu_4NBF_4 MeCN electrolyte solution contained *p*-AnOH (1 mmol, 0.124 mL) and 2,6-lutidine (5 mmol, 0.579 mL). (*) Unidentified solvent impurity.

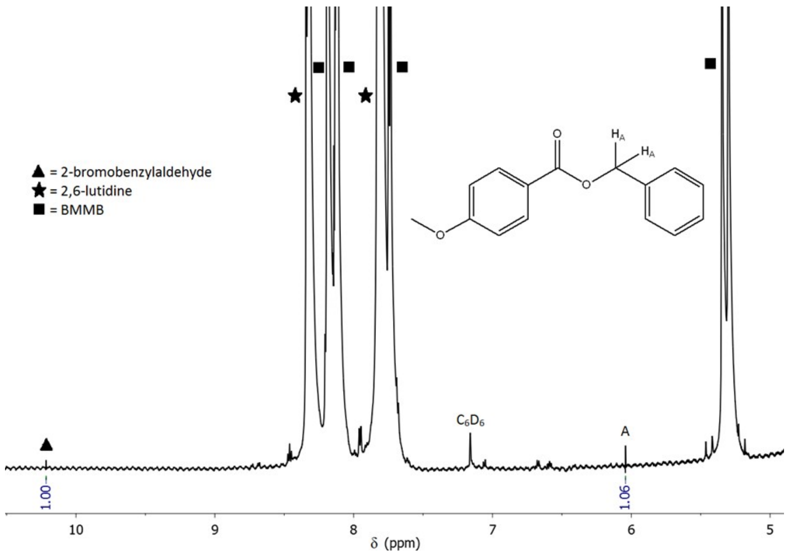

Figure S13. ^{1}H NMR spectrum of the electrolyte solution from a CPE oxidation of BMMB with **1**@GC, with internal 2-bromobenzaldehyde/C_6D_6 standard. The CPE experiment was run for 5 hours at 1.37 V vs. SCE using a 1 cm^2 GC plate modified with **1** as the working electrode. The 0.1 M Bu_4NBF_4 MeCN electrolyte solution contained BMMB (1 mmol, 0.228 mL), 2,6-lutidine (5 mmol, 0.579 mL) and H_2O (3.72 mmol, 0.067 mL).

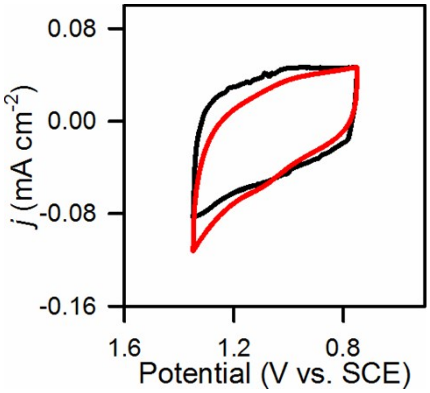

Figure S14. CVs of **1**@GC (black) before and (red) after bulk electrolysis. Following CPE experiments, the CPE cell was drained of electrolyte solution containing substrate via cannula transfer, rinsed once with fresh electrolyte solution then filled again with fresh electrolyte solution. A CV was then recorded. Decreased reversibility of the mediator after electrolysis is most likely due to oxidation of residual substrate. All CVs recorded under N_2 at 50 mV/s in 0.1 M Bu_4NBF_4 MeCN solution.

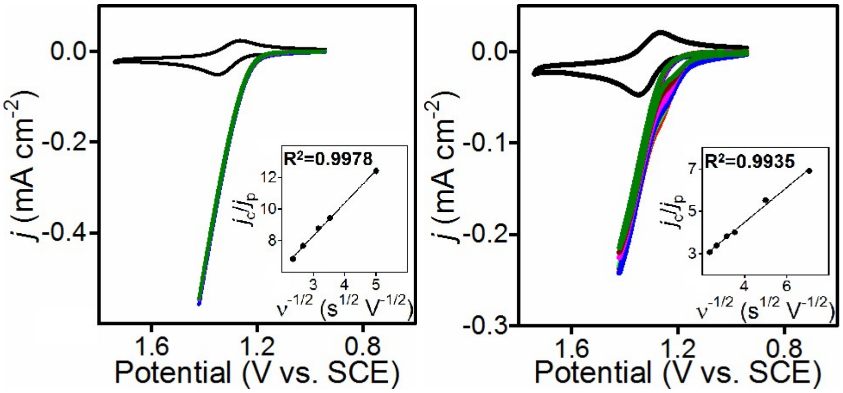

Figure S15. (left) CVs of 1.6 mM **1** in solution (black); and with 80 mM **2a**, 200 mM 2,6-lutidine at 180, 140, 100, 80 and 40 mV/s. **Inset**: Plot of j_c/j_p vs. inverse square root scan rate. (Right) CV of 1.6 mM **1** in solution (black); and with 80 mM **3a**, 200 mM 2,6-lutidine at 180, 140, 100, 80 and 40 mV/s. **Inset**: Plot of j_c/j_p vs. inverse square root scan rate. j_c and j_p values were recorded at 1.32 V vs. SCE. All CVs recorded in 0.1 M Bu_4NBF_4 MeCN solution.

References

[1] R. Francke and R. D. Little, *J. Am. Chem. Soc.,* 2014, **136**, 427-435.

[2] H. E. Gottlieb, V. Kotlyar and A. Nudelman, *J. Org. Chem.*, 1997, **62**, 7512.

[3] C. C. L. McCrory, A. Devadoss, X. Ottenwaelder, R. D. Lowe, T. D. P. Stack, C. E. D. Chidsey, *J. Am. Chem. Soc.*, 2011, **133**, 3696-3699.

[4] N. G. Connelly and W. E. Geiger, *Chem. Rev.*, 1996, **96**, 877.

[5] H. O. House, E. Feng and N. P. Peet, *J. Org. Chem.*, 1971, **36**, 2371.

[6] a) M. A. Pellow, T. D. Stack, C. Chidsey, *Langmuir*, 2013, **29**, 5383-5387. b) A. Devadoss, C. Chidsey, *J. Am. Chem. Soc.*, 2007, **129**, 5370-5371. c) M. V. Sheridan, K. Lam, W. E. Geiger, *Angew. Chem. Int. Ed.*, 2013, **52**, 12897-12900.
